# Multimorbidity and survival for patients with acute myocardial infarction in England and Wales: Latent class analysis of a nationwide population-based cohort

**DOI:** 10.1371/journal.pmed.1002501

**Published:** 2018-03-06

**Authors:** Marlous Hall, Tatendashe B. Dondo, Andrew T. Yan, Mamas A. Mamas, Adam D. Timmis, John E. Deanfield, Tomas Jernberg, Harry Hemingway, Keith A. A. Fox, Chris P. Gale

**Affiliations:** 1 Leeds Institute of Cardiovascular and Metabolic Medicine, University of Leeds, Leeds, United Kingdom; 2 Department of Medicine, University of Toronto, Toronto, Ontario, Canada; 3 Keele Cardiovascular Research Group, Keele University, Stoke-on-Trent, United Kingdom; 4 NIHR Cardiovascular Biomedical Research Unit, Barts Heart Centre, London, United Kingdom; 5 National Institute for Cardiovascular Outcomes Research, University College London, London, United Kingdom; 6 Department of Clinical Sciences, Danderyd Hospital, Karolinska Institutet, Stockholm, Sweden; 7 Farr Institute of Health Informatics Research, University College London, London, United Kingdom; 8 NIHR Biomedical Research Centre, University College London Hospitals NHS Foundation Trust, University College London, London, United Kingdom; 9 Centre for Cardiovascular Science, University of Edinburgh, Edinburgh, United Kingdom; 10 York Teaching Hospital NHS Foundation Trust, York, United Kingdom; National University of Singapore, SINGAPORE

## Abstract

**Background:**

There is limited knowledge of the scale and impact of multimorbidity for patients who have had an acute myocardial infarction (AMI). Therefore, this study aimed to determine the extent to which multimorbidity is associated with long-term survival following AMI.

**Methods and findings:**

This national observational study included 693,388 patients (median age 70.7 years, 452,896 [65.5%] male) from the Myocardial Ischaemia National Audit Project (England and Wales) who were admitted with AMI between 1 January 2003 and 30 June 2013. There were 412,809 (59.5%) patients with multimorbidity at the time of admission with AMI, i.e., having at least 1 of the following long-term health conditions: diabetes, chronic obstructive pulmonary disease or asthma, heart failure, renal failure, cerebrovascular disease, peripheral vascular disease, or hypertension. Those with heart failure, renal failure, or cerebrovascular disease had the worst outcomes (39.5 [95% CI 39.0–40.0], 38.2 [27.7–26.8], and 26.6 [25.2–26.4] deaths per 100 person-years, respectively). Latent class analysis revealed 3 multimorbidity phenotype clusters: (1) a high multimorbidity class, with concomitant heart failure, peripheral vascular disease, and hypertension, (2) a medium multimorbidity class, with peripheral vascular disease and hypertension, and (3) a low multimorbidity class. Patients in class 1 were less likely to receive pharmacological therapies compared with class 2 and 3 patients (including aspirin, 83.8% versus 87.3% and 87.2%, respectively; β-blockers, 74.0% versus 80.9% and 81.4%; and statins, 80.6% versus 85.9% and 85.2%). Flexible parametric survival modelling indicated that patients in class 1 and class 2 had a 2.4-fold (95% CI 2.3–2.5) and 1.5-fold (95% CI 1.4–1.5) increased risk of death and a loss in life expectancy of 2.89 and 1.52 years, respectively, compared with those in class 3 over the 8.4-year follow-up period. The study was limited to all-cause mortality due to the lack of available cause-specific mortality data. However, we isolated the disease-specific association with mortality by providing the loss in life expectancy following AMI according to multimorbidity phenotype cluster compared with the general age-, sex-, and year-matched population.

**Conclusions:**

Multimorbidity among patients with AMI was common, and conferred an accumulative increased risk of death. Three multimorbidity phenotype clusters that were significantly associated with loss in life expectancy were identified and should be a concomitant treatment target to improve cardiovascular outcomes.

**Trial registration:**

ClinicalTrials.gov NCT03037255.

## Abstract 2

Using latent class analysis, Marlous Hall and colleagues examine the associations between mulitmorbidity and survival in patients with acute myocardial infarction.

## Author summary

**Why was this study done?:**

- Survival following a heart attack (acute myocardial infarction) has improved over recent decades.
- The population is ageing, and there are more patients living longer with multiple long-term health conditions (multimorbidity).
- Previous studies have looked at the associations between individual long-term health conditions and survival following a heart attack, but little is known about patterns of multiple conditions and how such multimorbidity patterns may be associated with patients’ outcomes.

**What did the researchers do and find?:**

- A total of 693,388 patients who had a heart attack between 2003 and 2013 across England and Wales were included in the study.
- 59.5% of patients had at least 1 of the following long-term health conditions at the time of their heart attack: diabetes, chronic obstructive pulmonary disease or asthma, heart failure, renal failure, cerebrovascular disease (stroke), peripheral vascular disease, or hypertension.
- There were 3 common multimorbidity patient groups: (1) patients with high multimorbidity at the time of their heart attack, who tended to have heart failure as well as peripheral vascular disease and hypertension, (2) patients with medium multimorbidity at the time of their heart attack, who tended to have peripheral vascular disease and hypertension, and (3) patients with few co-morbidities.
- Patients who had high and medium levels of multimorbidity had average reduced life expectancies of 2.9 and 1.5 years, respectively, compared with patients who had few co-morbidities.

**What do these findings mean?:**

- This study shows that many patients with a heart attack have multimorbidity, and that those with high and medium levels of multimorbidity are likely to experience a significant reduction in years of life expectancy.
- Whilst this study included 7 conditions most commonly occurring in patients who have a heart attack, there may be other conditions including dementia and cancer that require investigation in this context.
- Future research should focus on developing treatment guidelines for the multimorbidity patient groups identified to improve outcomes for heart attack patients with multimorbidity.

## Introduction

The increasing prevalence of long-term health conditions, and consequent growing prevalence of multimorbidity (the presence of multiple co-morbidities), is a major global challenge facing healthcare systems [1,2]. Presently, around two-thirds of patients with cardiovascular disease are estimated to have at least 1 long-term health condition [3]. With improved survival rates following acute myocardial infarction (AMI) as well as an ageing population [4–6], there are more patients living longer with multimorbidity, which is associated with reduced quality of life, increased healthcare burden, and increased mortality [3,7,8].

Although many prior studies have assessed the association between individual co-morbidities—including diabetes [9,10], chronic obstructive pulmonary disease (COPD) [11–15], and heart failure [16]—and survival in patients with AMI, few have quantified the burden of multimorbidity—in particular how the complex patterns of multiple conditions simultaneously associate with mortality. Where data do exist on multimorbidity clusters, studies are limited to regional rather than national data and have relied on basic analytical techniques that consider composite additive [17,18] or weighted [19–22] co-morbidity scores, or focus on all possible combinations of conditions [18,23,24]. Moreover, previous data are mostly limited to short- (30 days) and medium-term (1 year) outcomes, with the exception of the study by Di Angelantonio et al. [24] (12.8 years of follow-up), even though such conditions are lifelong diseases and, therefore, warrant investigation of outcomes over the longer term. More sophisticated methods, such as latent class analysis, enable insights into multidimensional disease patterns based on probabilistic modelling of specific conditions without the aforementioned limitations [25]. Furthermore, insights into the association of multimorbidity with survival following AMI from latent class analysis may help define and target therapeutic strategies to specific groups of patients in an attempt to reduce premature death [1,26,27].

Therefore, this study aimed to investigate which multimorbidity phenotype clusters exist across a range of pre-existing long-term health conditions and study their association with long-term survival for patients hospitalised with AMI. We hypothesise that the presence of multimorbidity confers an increased long-term risk of death for patients with AMI. In addressing this hypothesis, we provide a greater understanding of the clustering of pre-existing conditions and their simultaneous burden on survival.

## Methods

### Ethical approval

The National Institute for Cardiovascular Outcomes Research, which includes the Myocardial Ischaemia National Audit Project (MINAP) database (Ref: NIGB ECC 1-06(d)/2011), has support under section 251 of the National Health Service Act 2006 to use patient information for medical research without informed consent. Further ethical approval, or patient consent, was not required under current National Health Service research governance arrangements, and all data analysed in the study were anonymised. This study is reported as per RECORD guidelines (S1 Checklist).

### Data and patients

Data for this study were obtained from MINAP, representing all hospitals in a single health system (the National Health Service in England and Wales). MINAP offers the opportunity to undertake population-based observational studies of an array of multimorbid conditions and their association with survival following AMI. Full details of MINAP have been published elsewhere [28]. The analytical cohort (*n =* 693,388) was drawn from 693,633 patients with AMI admitted to 1 of 247 hospitals between 1 January 2003 and 30 June 2013 (S1 Text; S1 Fig). Patients were eligible for the study if they were aged 18 years or over; where multiple admissions for AMI were recorded per person, only the first admission was included. We excluded 245 (0.04%) patients due to missing mortality data. Patients were defined as having multimorbidity if they had a history of 1 or more of the following conditions when admitted to hospital with AMI: diabetes mellitus, COPD or asthma, chronic heart failure, chronic renal failure (defined as creatinine chronically >200 μmol/l [>2.26 mg/dl]), cerebrovascular disease, peripheral vascular disease, or hypertension (defined as a patient already receiving treatment [drug, dietary, or lifestyle] for hypertension or with recorded blood pressure >140/90 mm Hg on at least 2 occasions prior to admission).

Patient-level data included baseline ischaemic risk (calculated using the Global Registry of Acute Coronary Events [GRACE] risk score parameters: age, cardiac arrest, electrocardiographic ST segment deviation, elevated cardiac enzymes, systolic blood pressure, heart rate on admission to hospital, prescription of a loop diuretic [substituted for Killip class] and creatinine [29,30]), patient demographics (sex and index of multiple deprivation [31]), type of AMI (ST-elevated myocardial infarction [STEMI] and non-ST-elevated myocardial infarction [NSTEMI]), medical history (smoking status, family history of coronary heart disease, hypertension, total cholesterol, previous AMI, angina, percutaneous coronary intervention, and coronary artery bypass graft surgery), pharmacological therapies at the time of discharge from hospital (aspirin, β-blockers, HMG-CoA reductase inhibitors [statins], ACE inhibitors/angiotensin receptor blockers, P2Y_12_ inhibitors, and aldosterone antagonists), revascularisation strategy (thrombolysis or coronary intervention [percutaneous coronary intervention or coronary artery bypass graft surgery] or both), and all-cause mortality (through patient-level linkage to the United Kingdom Office for National Statistics). Patients were followed up for mortality status to a censoring date of 30 April 2011 for those diagnosed between 2003 and 2009, and a final censoring date of 31 December 2013 for those diagnosed from 2010 onwards. This resulted in a maximum observed follow-up time of 8.4 years (median and interquartile range: 2.3, 0.9–4.0 years), representing 1,872,468 person-years at risk.

### Statistical analyses

While no formal analysis plan exists, all analytical methods described here were planned prospectively prior to analyses. Where data-driven approaches were used for model selection, or sensitivity analyses conducted as a result of peer review, this has been clearly indicated below.

Latent class analysis was performed to assimilate individual patient data for multiple long-term health conditions into multimorbidity classes. The resultant classes represented probabilistic groups of patients with similar combinations of conditions, and as such depicted complex patterns of higher order interactions between multiple conditions (S3 Text; S3 Fig; S4 Table). The latent class analysis was based only on the probability distributions of the baseline long-term health conditions and did not take outcomes into account. Latent class analysis was preferred over simpler techniques such as creating an additive score of long-term health conditions, through which the granularity of specific combinations of conditions would be lost, or analysing all possible combinations of conditions, which is subject to high false positive rates (type I errors) and can lead to low study power.

Baseline characteristics were described according to each multimorbidity phenotype cluster using numbers and percentages for categorical data and means and standard deviations and medians and interquartile ranges for normally and non-normally distributed continuous variables, respectively. The differences in baseline characteristics between multimorbidity phenotype clusters were summarised using chi-squared tests, *t* tests, and Wilcoxon rank-sum tests appropriate to the data type and distribution. Furthermore, differences in receipt of guideline-recommended therapies were compared according to multimorbidity phenotype cluster.

Royston–Parmar flexible parametric survival models [32] based on all-cause mortality were fitted to determine the association of multimorbidity phenotype clusters with long-term survival (8.4 years). In addition, the association of individual pre-existing conditions and the accumulation of multimorbid conditions (grouped into 0, 1, and 2 or more conditions) with long-term survival was assessed using the same flexible parametric survival models. Left ventricular ejection fraction (LVEF; categorised as good, ≥50%; moderate, 30%–49%; and poor, <30%) and estimated glomerular filtration rate (eGFR; categorised as normal or mild impairment, ≥60 ml/min per 1.73 m^2^; moderate impairment, 30–59 ml/min per 1.73 m^2^; or severe/very severe impairment, <30 ml/min per 1.73 m^2^) were used to model the association of the severity of chronic heart failure and chronic renal failure, respectively, with survival. The models were adjusted for known confounders based on clinical consideration and previous research [27,33] including baseline ischaemic risk, demographic variables, type of AMI, medical history, revascularisation strategy, and pharmacological therapies at discharge as defined earlier. We selected flexible parametric models a priori in favour of standard Cox regression to allow for modelling of non-proportional hazards as well as extension to a relative survival framework for calculation of loss in life expectancy (more details below). Further sensitivity analyses were conducted following peer review in order to assess the impact of the increase in recorded multimorbidity between 2003 and 2004 upon the results (S3 Table).

Finally, we estimated the loss of life expectancy in years compared with the age-, sex-, and year-matched populace of the UK [34] that was due to multimorbidity phenotype cluster as well as according to individual and cumulative conditions. Mortality data for England and Wales were obtained from Office for National Statistics life tables [35] (S4 Text).

Multiple imputation by chained equations was used to produce 10 imputed datasets to minimise potential bias due to missing data (S2 Text; S1 Table), using previously defined methods for imputation of the MINAP data [36]. Pooled estimates and accompanying 95% confidence intervals were generated according to Rubin’s rules [37]. The scale (proportional hazards, proportional odds, or normal) and complexity (number of degrees of freedom) for flexible parametric survival models were determined by minimising the Akaike information criterion and the Bayesian information criterion for the complete case analysis as well as for each individual imputation. According to good practice guidelines for multiple imputation, a sensitivity analysis comparing the main imputed analysis with a complete case analysis was conducted (S2 Table; S2 Fig). All tests were 2-sided, and statistical significance was considered as *P <* 0.05. Statistical analyses were performed in Stata MP version 14 (http://www.stata.com/), R version 3.1.2 (https://cran.r-project.org/), and Mplus version 7.3 (https://www.statmodel.com/).

## Results

**Fig 1:**
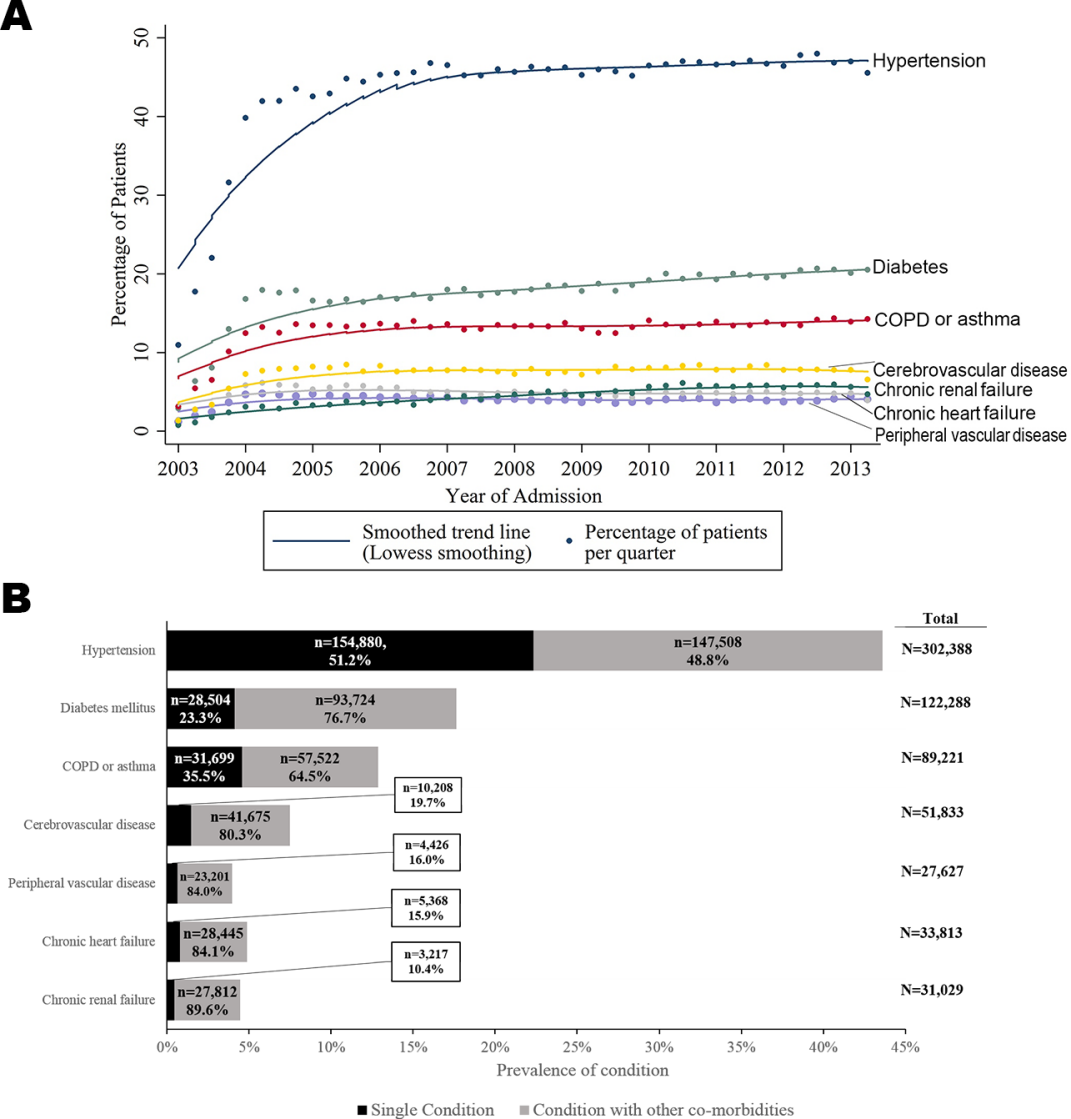
Time trends and prevalence of multimorbid conditions for patients hospitalised with acute myocardial infarction in England and Wales, 2003–2013. (A) Time trends. (B) Prevalence. COPD, chronic obstructive pulmonary disease.

There were a total of 693,388 patients included (median age 70.7 years; 452,896 [65.5%] men) and 1,872,468 person-years follow-up. There were 412,809 (59.5%) patients with AMI who had at least 1 pre-existing co-morbid condition. The majority of these had 1 condition (238,302, 57.7%), whereas 120,693 (29.2%) had 2, and 53,814 (13.0%) had 3 or more, up to a maximum of 7 (63, 0.02%). The most prevalent conditions were hypertension (*n =* 302,388, 45.9%), diabetes mellitus (*n =* 122,228, 18.6%), and COPD or asthma (*n =* 89,211, 13.6%). Whilst chronic renal failure, chronic heart failure, and peripheral vascular disease were the least prevalent, patients with these conditions most frequently had additional conditions (27,812 [89.6%], 28,445 [84.1%], and 23,201 [84.0%], respectively) (Fig 1).

**Fig 2:**
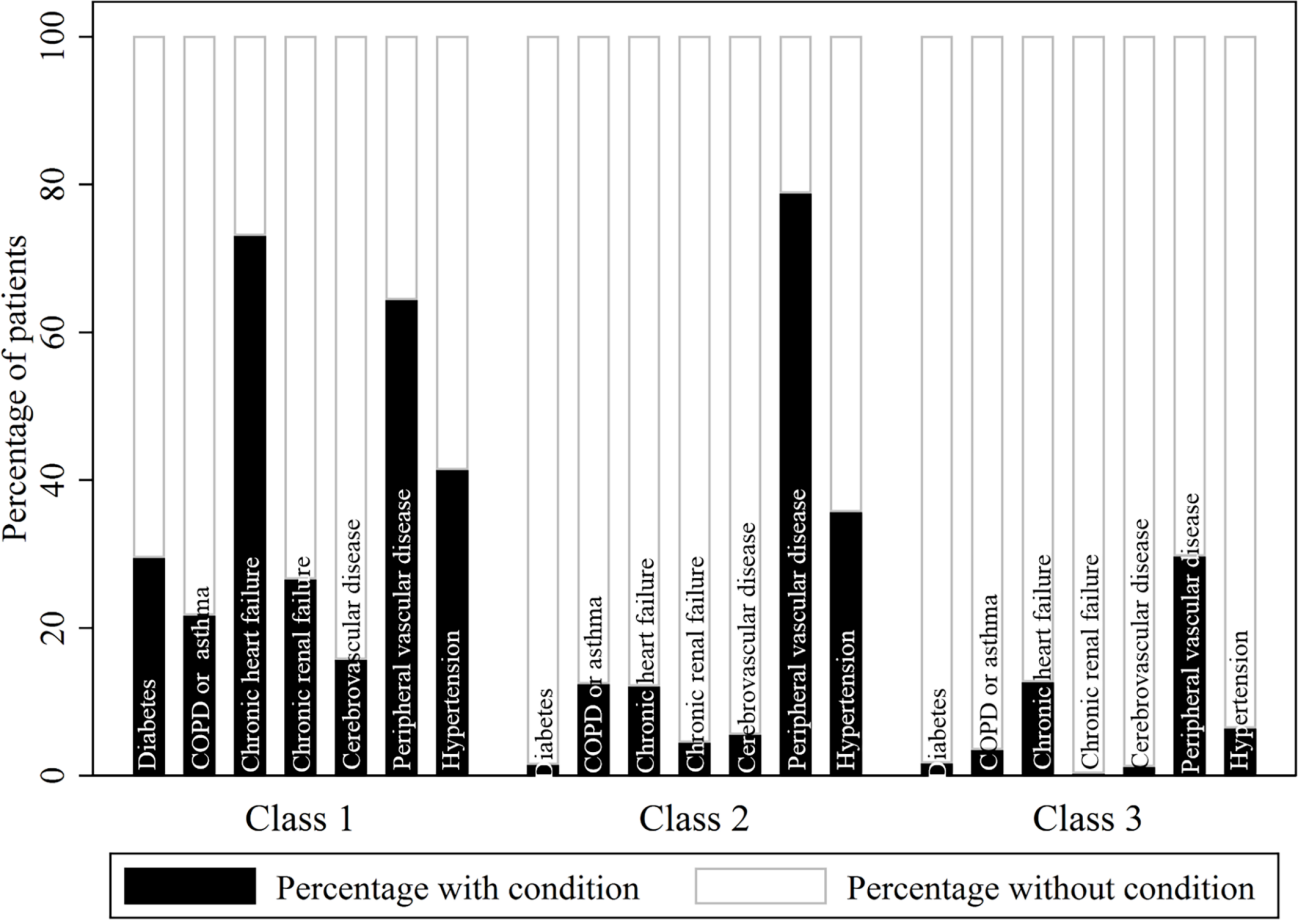
Probabilistic distribution of multimorbid conditions within each multimorbidity phenotype cluster. S5 Table provides the full probabilistic latent class structure. The observed proportions of patients with and without each condition per class indicate that class 1 characterises patients with high multimorbidity, especially including chronic heart failure, peripheral vascular disease, and hypertension; class 2 characterises patients with medium multimorbidity, especially including peripheral vascular disease and hypertension; and class 3 characterises patients with low multimorbidity but with peripheral vascular disease. COPD, chronic obstructive pulmonary disease.

Latent class analysis revealed 3 distinct groups of patients in terms of their multimorbidity profile: class 1, a high multimorbidity cluster, with concomitant heart failure, peripheral vascular disease, and hypertension; class 2, a medium multimorbidity cluster, with peripheral vascular disease and hypertension; and class 3, with low levels of multimorbidity overall but with peripheral vascular disease (Fig 2; S5 Table).

Patients in multimorbidity class 1 (high multimorbidity with concomitant heart failure, peripheral vascular disease, and hypertension) tended to be older (median age 78.9 years; IQR 71.0–84.8) than those in multimorbidity class 2 (medium multimorbidity with concomitant peripheral vascular disease and hypertension; median age 74.0 years; IQR 64.8–81.3) and multimorbidity class 3 (low levels of multimorbidity overall but with peripheral vascular disease; median age 68.5 years; IQR 57.5–79.0). Class 1 patients also tended to be female (40.5% versus 38.5% and 33.1%, respectively) and more often had NSTEMI (83.2%) than STEMI compared with class 2 and 3 patients (71.6% and 57.6%, respectively). Furthermore, patients in class 1 were less likely to receive pharmacological therapies after accounting for eligibility compared with class 2 and 3 patients (Table 1). Of note, there were more people in the high and medium multimorbidity phenotype clusters (class 1 and 2) in the latest period of the study compared with the earliest period (class 1: 9.0% in 2011–2013 versus 7.9% in 2003–2006; class 2: 16.6% in 2011–2013 versus 13.9% in 2003–2006). There was an associated 2.0% (95% CI 1.9%–2.3%) increase in the number of conditions per year (Fig 1).

**Table 1:** Baseline characteristics stratified according to multimorbidity phenotype cluster at the time of acute myocardial infarction hospitalisation, 2003–2013 (prior to multiple imputation for missing data).

Variable	Measure	Total cohort, *N =* 693,388	Multimorbidity phenotype cluster^1^			*P*-value^2^	Missing (%)^3^			
			Class 1 *N =* 47,839	Class 2 *N =* 87,009	Class 3 *N =* 433,215		Total cohort	Class 1	Class 2	Class 3
Age (years)^4^	Median (IQR)	70.7 (59.4–80.1)	78.9 (71–84.8)	74 (64.8–81.3)	68.5 (57.5–79.0)	<0.001	0.1	0.1	0.1	0.1
**Sex (male)**^**5**^	*N* (%)	452,896 (65.5)	28,613 (59.9)	53,412 (61.5)	288,948 (66.9)	<0.001	0.3	0.1	0.2	0.2
**Year of admission**^**6**^						<0.001	0	0	0	0
2003–2006	*N* (%)	244,499 (35.3)	14,040 (7.9)	24,764 (13.9)	139,769 (78.3)					
2007–2010	*N* (%)	274,085 (39.5)	19,748 (8.5)	36,458 (15.6)	177,605 (76.0)					
2011–2013	*N* (%)	174,804 (25.2)	14,051 (9.0)	25,787 (16.6)	115,841 (74.4)					
**Index of multiple deprivation**^**5**^	Median (IQR)	18.3 (10.6 to 31.8)	19.4 (11.3–33.5)	19.5 (11.2–33.7)	17.7 (10.3–30.6)	<0.001	8.2	7.9	7.5	8.0
**GRACE risk score**^**7**^						<0.001	56.0	45.6	46.6	50.5
<70	*N* (%)	21,686 (7.1)	258 (1.0)	1,707 (3.7)	18,606 (8.7)					
70–87	*N* (%)	33,539 (11.0)	576 (2.2)	3,539 (7.6)	27,785 (13.0)					
≥88	*N* (%)	249,892 (81.9)	25,187 (96.8)	41,231 (88.7)	167,975 (78.4)					
**Index AMI STEMI(versus NSTEMI)**	*N* (%)	274,220 (39.5)	8,019 (16.8)	24,721 (28.4)	183,601 (42.4)	<0.001	0	0	0	0
**SBP (mm Hg)**^4^	Mean (SD)	139.2 (29)	137.1 (30.6)	142.2 (29.9)	138.9 (28.4)	<0.001	19.3	10.3	10.5	11.3
**Heart rate (beat/min)**^4^	Mean (SD)	81.9 (23.2)	88.3 (24.6)	84.4 (23.2)	80.6 (22.6)	<0.001	19	10.1	10.3	10.9
**Total cholesterol (mmol/l)**^**8**^	Median (IQR)	5.0 (1.4)	4.2 (1.4)	4.4 (1.4)	5.2 (1.4)	<0.001	35.7	48.6	39.0	32.0
**Creatinine (μmol/l)**	Median (IQR)	91.0 (76.0–112.0)	131.0 (98.0–188.0)	97.0 (79.0–124.0)	88.0 (74.0–105.0)	<0.001	44.5	35.5	35.4	39.1
**Medical history**^**5**^										
Current or ex-smoker	*N* (%)	390,956 (62.2)	26,406 (60.6)	48,858 (59.9)	262,649 (64.0)	<0.001	9.4	8.9	6.2	5.2
Family history of coronary heart disease	*N* (%)	140,388 (32.7)	6,358 (21.1)	18,338 (30.0)	104,220 (33.8)	<0.001	38.2	37.0	29.6	28.8
Previous AMI	*N* (%)	136,482 (21.9)	20,620 (43.5)	25,791 (29.8)	74,360 (17.2)	<0.001	10.1	0.9	0.5	0.3
Previous angina	*N* (%)	169,454 (27.5)	23,547 (49.7)	33,206 (38.4)	94,389 (21.9)	<0.001	11.2	1.0	0.7	0.4
**Admission treatment**^**5**^										
Revascularisation^9^	*N* (%)	227,275 (42.1)	5,238 (14.8)	18,149 (26.9)	145,951 (42.7)	<0.001	22.2	26.2	22.5	21.1
Loop diuretic	*N* (%)	155,674 (28.1)	27,894 (62.9)	29,265 (36.8)	85,848 (22.0)	<0.001	20.1	7.3	8.7	10.0
**Discharge medication**^**5**^										
Aspirin	*N* (%)	510,387 (85.7)	31,780 (83.8)	6,384 (87.3)	329,292 (87.2)	<0.001	9.5	20.7	15.9	12.8
β-blocker	*N* (%)	427,191 (79.1)	23,443 (74.0)	54,013 (80.9)	280,719 (81.4)	<0.001	10.3	33.8	23.3	20.4
Statin	*N* (%)	503,686 (83.4)	31,718 (80.6)	64,379 (85.9)	324,934 (85.2)	<0.001	10.1	17.7	13.9	11.9
ACEi or ARB	*N* (%)	442,954 (77.5)	25,175 (74.7)	56,717 (80.6)	289,225 (79.4)	<0.001	10.7	29.5	19.1	15.9
P2Y_12_ inhibitor	*N* (%)	217,539 (94.4)	14,854 (94.9)	30,730 (95.0)	147,771 (94.8)	0.264	56.2	67.3	62.8	64.0
Aldosterone antagonist	*N* (%)	17,035 (10.3)	2,826 (21.8)	2,655 (11.1)	9,825 (8.8)	<0.001	60.0	72.9	72.6	74.1
**Mortality (unadjusted)**										
30 days	*N* (%)	62,950 (9.1)	8,139 (17.0)	8,656 (10.0)	32,002 (7.4)	<0.001	0	0	0	0
1 year	*N* (%)	102,254 (14.8)	19,025 (39.8)	18,600 (21.4)	62,151 (14.4)	<0.001	0	0	0	0
5 years	*N* (%)	204,667 (29.5)	27,471 (57.4)	29,537 (34.0)	97,161 (22.4)	<0.001	0	0	0	0

^1^Class 1 characterises patients with high multimorbidity, especially with concomitant chronic heart failure, peripheral vascular disease, and hypertension. Class 2 characterises patients with medium multimorbidity, especially peripheral vascular disease and hypertension. Class 3 characterises patients with low multimorbidity but with peripheral vascular disease.

^2^*P*-value for difference between classes.

^3^S6 Table contains patient characteristics for those with missing latent class data (prior to multiple imputation for missing data).

^4^Values are means and standard deviations, and *P*-values are derived from *t* tests.

^5^Values are numbers and percentages, and *P*-values are derived from chi-squared tests.

^6^Column percentages shown to highlight temporal trend.

^7^Given international guidelines, Global Registry of Acute Coronary Events (GRACE) risk score was categorised into lowest (<70), low (70 to 87), and intermediate-to-high risk (≥88).

^8^Values are medians and interquartile ranges, and *P*-values are derived from Wilcoxon rank-sum tests.

^9^Thrombolysis or coronary intervention (percutaneous coronary intervention or coronary artery bypass graft surgery) or both.

ACEi, angiotensin-converting enzyme inhibitor; AMI, acute myocardial infarction; ARB, angiotensin receptor blocker; NSTEMI, non-ST-elevated myocardial infarction; SBP, systolic blood pressure; STEMI, ST-elevated myocardial infarction.

### Outcomes

Unadjusted all-cause mortality was higher for those in class 1 than for those in classes 2 and 3 at 30 days (17.0% [95% CI 16.7%–17.4%] versus 10% [9.7%–10.1%] and 7.4% [7.3%–7.5%], respectively; *P <* 0.001), 1 year (39.8% [39.3%–40.2%] versus 21.4% [21.1%–21.6%] and 14.4% [14.2%–14.5%]; *P <* 0.001), and 5 years (57.4% [57.0%–57.9%] versus 34.0% [33.6%–34.3%] and 22.4% [22.3%–22.6%]; *P <* 0.001).

Those in the high multimorbidity cluster with chronic heart failure, peripheral vascular disease, and hypertension (class 1) had a 2.4-fold increased hazard of death compared with low-multimorbidity patients (class 3) (hazard ratio [HR] 2.40; 95% CI 2.33–2.47) over the 8.4-year follow-up period. Those who were in the medium multimorbidity cluster with peripheral vascular disease and hypertension (class 2) had a 1.5-fold increased hazard compared with those with low levels of multimorbidity (class 3) (HR 1.45; 95% CI 1.41–1.48) (Table 2).

**Table 2:** Unadjusted and adjusted HRs for long-term survival according to multimorbidity class or health condition obtained from flexible parametric survival models after multiple imputation (5 degrees of freedom, odds scale).

Multimorbidity or long-term health condition	Deaths per 100 person-years (95% CI)	Median time to death in days (IQR)	Unadjusted HR (95% CI) or *P*-value	Adjusted HR (95% CI)^1^ or *P*-value
**Multimorbidity phenotype cluster**^2^			***P <* 0.001**	***P <* 0.001**
Class 1	36.7 (36.6–37.2)	138.9 (21.0–499.7)	4.20 (4.13–4.28)	2.40 (2.33–2.47)
Class 2	15.5 (15.4–15.7)	197.9 (27.4–657.5)	1.80 (1.78–1.83)	1.45 (1.41–1.48)
Class 3	8.6 (8.5–8.6)	176.9 (15.0–696.5)	1 (ref)	1 (ref)
**Diabetes mellitus**			***P <* 0.001**	***P <* 0.001**
Yes	16.6 (16.5–16.8)	189.9 (21.9–657.5)	1.68 (1.65–1.70)	1.21 (1.19–1.24)
No	9.9 (9.8–9.9)	175.9 (15.0–705.5)	1 (ref)	1 (ref)
**COPD or asthma**			***P <* 0.001**	***P <* 0.001**
Yes	17.2 (17.0–17.4)	186.3 (18.3–657.5)	1.68 (1.66–1.71)	1.17 (1.14–1.19)
No	10.2 (10.1–10.2)	176.9 (15.0–701.5)	1 (ref)	1 (ref)
**Chronic heart failure**			***P <* 0.001**	***P <* 0.001**
Yes	39.5 (39.0–40.0)	131.5 (18.3–518.7)	3.94 (3.86–4.03)	1.87 (1.81–1.93)
No	10.0 (9.9–10.0)	189.9 (16.0–719.5)	1.86 (1.70–2.04)	1.91 (1.64–2.22)
**LVEF**			***P <* 0.001**	***P <* 0.001**
Moderate (30%–49%)	33.0 (31.5–34.6)	159.9 (42.9–484.7)	1.21 (1.10–1.33)	1.27 (1.10–1.47)
Poor (<30%)	45.1 (43.1–47.0)	94.9 (19.0–356.8)	1.86 (1.70–2.04)	1.91 (1.64–2.22)
Good (≥50%)	28.8 (27.1–30.5)	190.9 (54.0–514.6)	1 (ref)	1 (ref)
**Chronic renal failure**			***P <* 0.001**	***P <* 0.001**
Yes	38.2 (37.7–38.8)	116.9 (18.3–449.3)	3.41 (3.34–3.48)	1.73 (1.67–1.79)
No	10.2 (10.1–10.2)	190.9 (16.0–725.5)	1 (ref)	1 (ref)
**eGFR (ml/min per 1.73 m**^**2**^**)**			***P <* 0.001**	***P <* 0.001**
Moderate impairment (30–59)	33.5 (32.5–34.5)	135.9 (23.0–477.7)	1.70 (1.51–1.92)	1.47 (1.25–1.74)
Severe/very severe impairment (<30)	54.4 (53.0–55.8)	95.9 (13.0–402.7)	2.72 (2.41–3.08)	2.01 (1.71–2.37)
Normal or mild impairment (≥60)	20.6 (19.1–22.1)	154.9 (25.0–506.7)	1 (ref)	1 (ref)
**Cerebrovascular disease**			***P <* 0.001**	***P <* 0.001**
Yes	26.6 (25.2–26.9)	142.5 (14.6–573.4)	2.71 (2.66–2.75)	1.77 (1.72–1.82)
No	10.0 (9.9–10.0)	186.9 (16.0–714.5)	1 (ref)	1 (ref)
**Peripheral vascular disease**			***P <* 0.001**	***P <* 0.001**
Yes	22.7 (22.3–23.1)	175.3 (18.3–628.2)	2.15 (2.10–2.20)	1.41 (1.36–1.46)
No	10.6 (10.5–10.6)	179.9 (16.0–697.5)	1 (ref)	1 (ref)
**Hypertension**			***P <* 0.001**	***P <* 0.001**
Yes	12.5 (12.4–12.6)	194.0 (19.0–698.0)	1.28 (1.27–1.30)	1.03 (1.02–1.06)
No	9.6 (9.5–9.7)	163.0 (14.0–688.0)	1 (ref)	1 (ref)
**Cumulative number of conditions**			***P <* 0.001**	***P <* 0.001**
1	10.7 (10.6–10.7)	181.8 (16.0–698.5)	1.69 (1.67–1.72)	1.32 (1.29–1.34)
2 or more	20.0 (19.9–20.2)	173.9 (20.8–603.6)	3.29 (3.25–3.34)	1.98 (1.94–2.03)
None	6.4 (6.4–6.5)	179.9 (12.0–783.5)	1 (ref)	1 (ref)

^1^Adjusted for sex, year of admission, index of multiple deprivation (continuous), Global Registry of Acute Coronary Events (GRACE) risk score (categorised into lowest [<70], low [70 to 87], and intermediate-to-high risk [≥88]), type of AMI (ST-elevated myocardial infarction versus non-ST-elevated myocardial infarction), smoking status, family history of coronary heart disease, history of hypertension, previous AMI, previous percutaneous coronary intervention, serum cholesterol (continuous), revascularisation (thrombolysis or coronary intervention [percutaneous coronary intervention or coronary artery bypass graft surgery] or both), and discharge medications (aspirin, β-blockers, ACE inhibitors/angiotensin receptor blockers, statins, P2Y_12_ inhibitors, and aldosterone antagonists).

^2^Class 1 characterises patients with high multimorbidity, especially with concomitant chronic heart failure, peripheral vascular disease, and hypertension, Class 2 characterises patients with medium levels of multimorbidity, especially peripheral vascular disease and hypertension. Class 3 characterises patients with low levels of multimorbidity but with peripheral vascular disease.

AMI, acute myocardial infarction; COPD, chronic obstructive pulmonary disease; eGFR, estimated glomerular filtration rate; HR, hazard ratio; LVEF, left ventricular ejection fraction.

**Fig 3:**
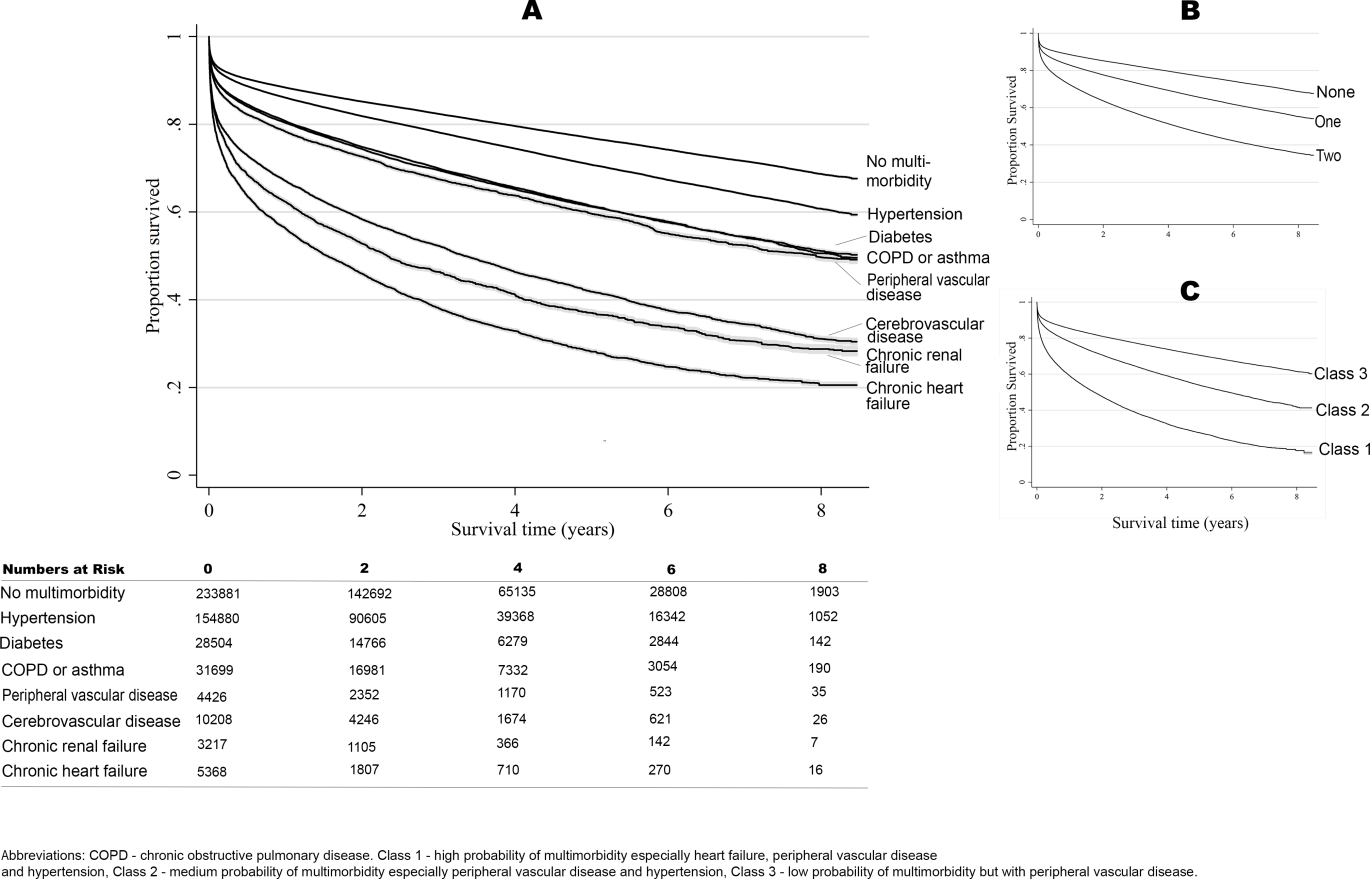
Long-term survival according to multimorbidity phenotype. Unadjusted Kaplan–Meier curves according to individual conditions (A), cumulative number of conditions (B), and multimorbidity phenotype cluster of multimorbid conditions (C).

For individual conditions, the worst outcomes were observed in patients with chronic heart failure (8.4-year mortality rate 63.5%, 95% CI 63.0%–64.1%; adjusted HR 1.87, 95% CI 1.81–1.93; median time to death 131.49 days, IQR 18.3–518.7; 39.5 deaths per 100 person years [/100 py]), chronic renal failure (8.4-year mortality rate 57.2%, 95% CI 56.7%–57.8%; adjusted HR 1.73, 95% CI 1.67–1.79; median time to death 116.9 days, IQR 18.3–449.3; 38.2 deaths/100 py), and cerebrovascular disease (8.4-year mortality rate 50.7%, 95% CI 50.3%–51.1%; adjusted HR 1.77, 95% CI 1.72–1.82; median time to death 142.5 days, IQR 14.6–573.4; 26.6 deaths/100 py) (Fig 3; Table 2). There was a corresponding decline in prognosis with decreasing LVEF (HR 1.27 [95% CI 1.10–1.47] and 1.91 [1.64–2.22] for moderate and poor LVEF, respectively, compared with good LVEF) and eGFR (HR 1.47 [1.25–1.74] and 2.01 [1.71–2.37] for moderate and severe/very severe impairment, respectively, compared with mild impairment or normal eGFR). Moreover, after adjustment for confounders, the accumulation of multimorbid conditions for patients with AMI was significantly associated with worse survival (HR 1.32 [95% CI 1.29–1.34] for 1 condition and HR 1.98 [1.94–2.03] for 2 or more conditions versus no multimorbidity; *P*-value for trend < 0.001).

The association between individual long-term health conditions and survival translated into a significant loss in life expectancy, such that those with chronic heart failure, chronic renal failure, and peripheral vascular disease had a loss in life expectancy of 2.91 (95% CI 2.58–3.25), 2.78 (95% CI 2.47–3.10), and 2.14 (95% CI 1.86–2.42) years, respectively, compared to those without long-term conditions (S7 Table). Moreover, multimorbidity was also associated with significant loss in life expectancy, such that those in class 1 had a loss in life expectancy of 2.89 years (95% CI 2.59–3.19), and those in class 2 a loss in life expectancy of 1.52 years (95% CI 1.33–1.71), compared with class 3 patients.

## Discussion

In this nationwide study of patients hospitalised with AMI, almost two-thirds had multimorbidity, most commonly with hypertension and diabetes mellitus. Those with 1 long-term health condition in addition to AMI were 32% more likely to die over the 8.4-year follow-up period, whereas those with 2 or more long-term health conditions were twice as likely to die, compared with those without multimorbidity. Each condition was associated with a unique and significant loss of life expectancy, which was greatest for those with chronic heart failure. Using latent class analysis, we identified 3 patient groups based on their probability of long-term health conditions that had distinct survival trajectories and may be considered as novel post-AMI survival phenotypes.

Global population demographics have changed such that nowadays patients with AMI are older and have more co-morbidities [38,39]. This and the fact that mortality rates from AMI have declined have created a new cohort of patients with multimorbidity who are now living with the aftermath of AMI [27]. In the United States, this amounts to over 85 million people (and 7 million in the United Kingdom) living with cardiovascular disease, of whom the majority are elderly or have co-existent long-term health conditions [40,41]. The resultant healthcare utilisation and associated direct costs are high and escalate according to multimorbidity [42,43]. A key international healthcare priority, therefore, is the reduction in multimorbidity and improvement in disease-free survival. To date, however, studies reporting multimorbidity have either focused on individual conditions [9–11,13,16] or have been largely limited to regional data with short-term outcomes [18,23]. Our study clearly depicts, in a modern healthcare system, the considerable burden of multimorbidity among patients with AMI and, importantly, identified clear patient classes for clusters of such conditions, such that multidisciplinary interventions may be targeted towards those in greatest need.

We noted that patients with AMI who also had a history of chronic heart failure, peripheral vascular disease, and hypertension had the worst prognosis. Such patients lived, on average, for about 4.5 months after hospitalisation and, after standardisation to population mortality rates, were estimated to have lost up to 5.5 years of life. Others have reported the detrimental effects of heart failure, yet the paucity of evidence for heart failure and multimorbidity is acknowledged [44]. Few have quantified the association of heart failure with prognosis after AMI [16,45], and, to our knowledge, none have identified the poor prognosis after AMI in combination with other co-morbidities within a national and contemporary dataset. Patients with cerebrovascular disease and chronic renal failure also had very poor outcomes, living on average 3 to 4 months after hospitalisation with AMI. Moreover, there was a cumulative deficit in survival among patients with more than 1 long-term health condition, such that those with 2 or more conditions in addition to AMI were twice as likely to die as those with none of the pre-existing conditions, and this level of multimorbidity was associated with between 1.9 and 2.6 expected years of life lost.

The identification of specific survival trajectories for phenotype clusters of multimorbidity has potentially important repercussions. This is because the proportion of patients with only 1 condition was lower than that for multiple conditions, and multimorbidity was more prevalent in the later years of the study. Future healthcare demands are therefore likely to arise from post-AMI patients with multimorbidity. To account for all possible multimorbid disease combinations and to investigate whether unique multimorbid phenotypes existed, we employed latent class analysis. This data-driven technique identified 3 significantly different disease-determined prognostic groups (so called ‘computational phenotypes’) [46]. Understanding the components of these classes of disease survivorship may help refine approaches to healthcare and stimulate the development of innovative health technologies aimed at improving clinical outcomes.

Although we found that patients with multimorbidity were less likely to receive guideline-indicated care, after adjustment for this, as well as patient demographics, these patients continued to have a significantly poorer prognosis. This suggests that even though outcomes could be improved to an extent through the greater provision of evidence-based in-hospital care, additional and/or novel interventions are warranted in this vulnerable population. For example, for heart failure and AMI, the design of new pharmacotherapies or greater use of community-based interventions such as homecare and follow-up visits may be important. It is worth noting that medical research designs such as randomised trials are optimised to focus on single diseases and single disease pathways: our findings suggest the importance of developing appropriate research designs for people with multiple diseases.

Our analyses are likely to have underestimated the association of multimorbidity with survival following AMI. This is because (1) some long-term health conditions may have been under-recorded in MINAP and (2) we did not have information about other diseases such as chronic arthritis, mental illness, dementia, obesity, cancer, and inflammatory bowel disease. However, in the calculation of years of life lost, we standardised by age, sex, and year to population mortality rates to ascertain relative survival. This technique allowed estimation of the association of index AMI with survival. On the other hand, it is possible that some patients may have had end-stage diseases, multiple intractable conditions, or frailty, whereby it may not have been appropriate to escalate care [47], and end-of-life decisions may have been commenced.

### Strengths and limitations

To our knowledge, MINAP is the largest nationwide single healthcare system database covering a prospective cohort of acute coronary syndromes. MINAP is designed to be representative of the management of acute coronary syndromes in a clinical setting, and our previous work has shown results consistent with those produced by randomised clinical trial data replicated in a real-world clinical setting [27]. Previous studies assessing a number of conditions have tended to rely on basic analytical techniques that either consider conditions independently or investigate all possible combinations of conditions [18,23]. Such techniques result in high rates of false positives (type I errors) and suffer from low statistical power. This study used latent class analysis to provide further insight into multidimensional disease patterns using a data-driven, probabilistic modelling approach. This allowed us to model the complex disease interactions of multiple conditions and their association with survival in a more sophisticated manner and without the aforementioned limitations. Despite these strengths, there were other study limitations. First, the study was reliant upon the accurate recording of data, and MINAP does not have 100% case ascertainment. Second, missing data, in particular missing data for each of the multimorbid conditions, could have biased the estimates. However, a thorough imputation strategy, including for multimorbid conditions, was implemented to minimise bias following a previous comprehensive study of the nature of missing data within MINAP [36]. Third, the study was limited to all-cause mortality due to the lack of available cause-specific mortality data. However, it has been shown that cause-specific mortality data may not always be reliable for cardiovascular-related causes of death [48]. Fourth, the study included historical data ranging from 2003 to 2013, which may therefore underestimate the most recent survival rates, due to improved treatments over time. Moreover, there was an apparent increase in the number of multimorbid conditions from 2003 to 2004, which may be a result of improved recording following the introduction of the quality outcomes framework in 2004, which incentivised general practitioners to screen for and identify co-morbid conditions, or a result of improved case ascertainment in this time period for patients who had NSTEMI, amongst whom multimorbidity tends to be more common. Fifth, the observational nature of the study means that we cannot demonstrate causation, though adjustment was made for confounders based on a rich set of available information in the study dataset and informed by external information from other studies.

### Conclusions

Among patients hospitalised with AMI, pre-existing multimorbid conditions were common and significantly associated with reduced survival. In particular, the presence of chronic heart failure, cerebrovascular disease, and chronic renal failure independently conferred the greatest risk of mortality and highest expected years of life lost. Three novel computational phenotypes of survivorship according to multimorbidity were identified, which may direct future research into the development of new pharmacotherapies and health service interventions for those in greatest need. Addressing multimorbidity among patients hospitalised with AMI is a necessary step in the international effort to reduce the burden of cardiovascular disease.

## Supporting information

S1 RECORD Checklist RECORD checklist. (DOCX)

S1 Fig Derivation of the analytical cohort from the MINAP dataset. (TIF)

S2 Fig Long-term survival according to multimorbidity phenotype: complete case analysis Unadjusted Kaplan–Meier curves according to individual conditions (A) and cumulative number of conditions (B) and according to multimorbidity phenotype cluster (C). (TIF)

S3 Fig Plot of the log likelihood for the different latent class solutions. (TIF)

S1 Table Imputation model specification. (DOCX)

S2 Table Complete case sensitivity analysis—unadjusted and adjusted flexible parametric survival models per condition (5 degrees of freedom, odds scale). (DOCX)

S3 Table Time period sensitivity analysis—unadjusted and adjusted flexible parametric survival models per condition (5 degrees of freedom, odds scale) for the period 2004–2013 (excluding 2003). (DOCX)

S4 Table Model fit statistics for latent class solutions. (DOCX)

S5 Table Conditional probabilities of the latent class structure. (DOCX)

S6 Table Patient characteristics for those with missing latent class data (prior to multiple imputation for missing data). (DOCX)

S7 Table Loss of life expectancy and 95% confidence intervals by long-term condition and age at hospitalisation for patients with AMI between 2003 and 2013 compared with the age-, sex-, and year-matched population of England and Wales. (DOCX)

S1 Text Data access and data cleaning. (DOCX)

S2 Text Multiple imputation analyses. (DOCX)

S3 Text Latent class analysis. (DOCX)

S4 Text Loss of life expectancy. (DOCX)

## Abbreviations

AMI: acute myocardial infarction
COPD: chronic obstructive pulmonary disease
eGFR: estimated glomerular filtration rate
HR: hazard ratio
LVEF: left ventricular ejection fraction
MINAP: Myocardial Ischaemia National Audit Project
NSTEMI: non-ST-elevated myocardial infarction
STEMI: ST-elevated myocardial infarction
