# Supplementary material for: Multimorbidity and survival for patients with acute myocardial infarction in England and Wales: Latent class analysis of a nationwide population-based cohort
Source: PLoS Med. 2018 Mar 6;15(3):e1002501. doi: 10.1371/journal.pmed.1002501 (PMC5839532; doi:10.1371/journal.pmed.1002501)
Supplement: S1 RECORD Checklist — (DOCX) [file pmed.1002501.s001.docx]

**S1 RECORD CHECKLIST:** RECORD Checklist

|  | **Item No.** | **STROBE items** | **RECORD items** | **Location in manuscript where items are reported** |
| --- | --- | --- | --- | --- |
| **Title and abstract** | | | | |
|  | 1 | (a) Indicate the study’s design with a commonly used term in the title or the abstract (b) Provide in the abstract an informative and balanced summary of what was done and what was found | RECORD 1.1: The type of data used should be specified in the title or abstract. When possible, the name of the databases used should be included.  RECORD 1.2: If applicable, the geographic region and timeframe within which the study took place should be reported in the title or abstract.  RECORD 1.3: If linkage between databases was conducted for the study, this should be clearly stated in the title or abstract. | Title: includes “nationwide population-based cohort”.  Abstract methods contains database name (MINAP).  Geographic region: ‘England & Wales’ reported in abstract methods. |
| **Introduction** | | | | |
| Background rationale | 2 | Explain the scientific background and rationale for the investigation being reported | | Introduction, Paragraph 1 “…growing prevalence of multimorbidity (the presence of multiple co-morbidities) is a major global challenge facing healthcare systems” |
| Objectives | 3 | State specific objectives, including any prespecified hypotheses | | Introduction, Paragraph 3 “…this study aimed to investigate which multimorbidity phenotype clusters exist across a range of pre-existing long-term health conditions and study their association with long-term survival for patients hospitalised with AMI. We hypothesise that the presence of multimorbidity confers an increased long-term risk of death for patients with AMI. In addressing this, we provide a greater understanding of the clustering of pre-existing conditions and their simultaneous burden on survival.” |
| **Methods** | | | | |
| Study Design | 4 | Present key elements of study design early in the paper | | Methods, Paragraph 3 – population based national observational study. |
| Setting | 5 | Describe the setting, locations, and relevant dates, including periods of recruitment, exposure, follow-up, and data collection | | Methods, Paragraph 3 - detailed study information is provided in the methods including “…AMI admitted to one of 247 hospitals between 1st January, 2003 and 30th June, 2013” and in Methods, Paragraph 4 “Patients were followed up for mortality status to a censoring date of 30th April 2011 for those diagnosed between 2003 and 2009, and a final censoring date of 31st December 2013 for those diagnosed from 2010 onwards. This resulted in a maximum observed follow up time of 8.4-years (median and interquartile range: 2.3, 0.9–4.0 years), representing 1,872,468 person-years at risk.” |
| Participants | 6 | *(a) Cohort study* - Give the eligibility criteria, and the sources and methods of selection of participants. Describe methods of follow-up  *Case-control study* - Give the eligibility criteria, and the sources and methods of case ascertainment and control selection. Give the rationale for the choice of cases and controls  *Cross-sectional study* - Give the eligibility criteria, and the sources and methods of selection of participants  *(b) Cohort study* - For matched studies, give matching criteria and number of exposed and unexposed  *Case-control study* - For matched studies, give matching criteria and the number of controls per case | RECORD 6.1: The methods of study population selection (such as codes or algorithms used to identify subjects) should be listed in detail. If this is not possible, an explanation should be provided.  RECORD 6.2: Any validation studies of the codes or algorithms used to select the population should be referenced. If validation was conducted for this study and not published elsewhere, detailed methods and results should be provided.  RECORD 6.3: If the study involved linkage of databases, consider use of a flow diagram or other graphical display to demonstrate the data linkage process, including the number of individuals with linked data at each stage. | The method of population selection is identified via a flow-diagram (S1 Fig), and exclusions also listed in Methods, Paragraph 3.  RECORD 6.2 and 6.3 N/A. |
| Variables | 7 | Clearly define all outcomes, exposures, predictors, potential confounders, and effect modifiers. Give diagnostic criteria, if applicable. | RECORD 7.1: A complete list of codes and algorithms used to classify exposures, outcomes, confounders, and effect modifiers should be provided. If these cannot be reported, an explanation should be provided. | Exposures (multimorbidity): Methods, Paragraph 3:  “Patients were defined as multimorbid if they had a history of any of the following conditions when admitted to hospital with AMI: diabetes mellitus, chronic obstructive pulmonary disease (COPD) or asthma, chronic heart failure, chronic renal failure (defined as creatinine chronically >200 µmol/L or >2.26 mg/dL), cerebrovascular disease, peripheral vascular disease, or hypertension (defined as a patient already receiving treatment (drug, dietary or lifestyle) for hypertension or with recorded BP > 140/90 on at least two occasions prior to admission).” MINAP records patients as having the conditions listed above at time of hospital admission (yes/no), therefore, no coding lists or algorithms were used to derive these.  Confounders: Methods, Paragraph 7 “The models were adjusted for known confounders based on clinical consideration and previous research including baseline ischaemic risk, demographic variables, AMI phenotype, medical history, revascularisation strategy, and pharmacological therapies at discharge as defined earlier” (Methods, Paragraph 4 has full list).”  Outcomes: Methods, Paragraph 7 – “A Royston-Parmar flexible parametric survival model based on **all-cause mortality** was fitted to determine the impact of individual pre-existing conditions as well as the cumulative effect of them (grouped into none, one condition and two or more conditions) upon **long-term survival (8.4 years)**.” |
| Data sources/ measurement | 8^*^ | For each variable of interest, give sources of data and details of methods of assessment (measurement).  Describe comparability of assessment methods if there is more than one group | | N/A |
| Bias | 9 | Describe any efforts to address potential sources of bias | | Missing data, Methods, Paragraph 9 “Multiple imputation by chained equations…” and Discussion, Paragraph 7 “Missing data, in particular missing data for each of the multimorbid conditions, could have biased the estimates. However, a thorough imputation strategy, including for multimorbid conditions, was implemented to minimise bias following a previous comprehensive study of the nature of missing data within MINAP”. |
| Study size | 10 | Explain how the study size was arrived at | | Methods, Paragraph 4 & S1 Fig.  “The analytical cohort (n=693,388) was drawn from 693,633 patients with AMI admitted to one of 247 hospitals between 1st January, 2003 and 30th June, 2013 (S1 Fig).” |
| Quantitative variables | 11 | Explain how quantitative variables were handled in the analyses. If applicable, describe which groupings were chosen, and why | | Table 2 footnote explains all variables adjusted in the modelling as well as their form (continuous/categorical and which categories)  “[Models are] adjusted for sex, year of admission, index of multiple deprivation (continuous), GRACE risk score (categorised into lowest (<70), low (70 to 87) and intermediate-to-high risk (≥88)), phenotype (ST-elevation myocardial infarction vs. non-ST-elevation myocardial infarction), smoking status, family history of coronary heart disease, history of hypertension, previous myocardial infarction, previous percutaneous coronary intervention, serum cholesterol (continuous), revascularisation (thrombolysis or coronary intervention (PCI or CABG) or both) and discharge medications (aspirin, β-blocker, ACEi/ARBs, statins, P2Y_12_ inhibitors, aldosterone antagonist).” |
| Statistical methods | 12 | (a) Describe all statistical methods, including those used to control for confounding  (b) Describe any methods used to examine subgroups and interactions  (c) Explain how missing data were addressed  (d) *Cohort study* - If applicable, explain how loss to follow-up was addressed  *Case-control study* - If applicable, explain how matching of cases and controls was addressed  *Cross-sectional study* - If applicable, describe analytical methods taking account of sampling strategy  (e) Describe any sensitivity analyses | | a) Methods, Paragraphs 5-9 details all statistical analyses, including confounders. “The models were adjusted for known confounders based on clinical consideration and previous research[27,33] including baseline ischaemic risk, demographic variables, AMI phenotype, medical history, revascularisation strategy, and pharmacological therapies at discharge as defined earlier. We selected flexible parametric models *apriori* in favour of standard Cox regression to allow for modelling of non-proportional hazards as well as extension to a relative survival framework to allow for calculation of the loss in life expectancy.”  b) Methods, Paragraph 7 - severity of chronic heart and renal failure. “Left ventricular ejection fraction (LVEF; categorised as good ≥50%, moderate 30-49% and poor <30%) and estimated glomerular filtration rate (eGFR; categorised as normal or mild ≥60 mL/min per 1.73m2, moderate 30-59 mL/min per 1.73m2 or severe to very severe <30 mL/min per 1.73m2) were used to respectively model the severity of chronic heart failure and chronic renal failure on survival”  c) Methods, Paragraph 9 – “Multiple imputation by chained equations was used to produce ten imputed datasets to minimise potential bias due to missing data (S2 Text, S1 Table), using previously defined methods for imputation of the Myocardial Ischaemia National Audit Project data…”  d) N/A  e) Complete cases analyses – S2 Fig, S2 Table & removal of 2003 from cohort, S3 Table. |
| Data access and cleaning methods |  | RECORD 12.1: Authors should describe the extent to which the investigators had access to the database population used to create the study population.  RECORD 12.2: Authors should provide information on the data cleaning methods used in the study. | | S1 Text/S1 Fig details the derivation of the analytical cohort and details of the database population to which the authors had access.  Data cleaning methods are described in S1 Text. |
| Linkage |  | RECORD 12.3: State whether the study included person-level, institutional-level, or other data linkage across two or more databases. The methods of linkage and methods of linkage quality evaluation should be provided. | | Methods, Paragraph 4 specifies that all-cause mortality was obtained through “patient-level linkage to the United Kingdom Office for National Statistics)” As described in S1 Text, data were linked at source prior to authors obtaining access. No details of linkage quality were available to the authors. |
| **Results** | | | | |
| Participants | 13 | (a) Report the numbers of individuals at each stage of the study (*e.g.*, numbers potentially eligible, examined for eligibility, confirmed eligible, included in the study, completing follow-up, and analysed)  (b) Give reasons for non-participation at each stage.  (c) Consider use of a flow diagram | RECORD 13.1: Describe in detail the selection of the persons included in the study (*i.e.,* study population selection) including filtering based on data quality, data availability and linkage. The selection of included persons can be described in the text and/or by means of the study flow diagram. | Flow diagram S1 Fig. |
| Descriptive data | 14 | (a) Give characteristics of study participants (*e.g.*, demographic, clinical, social) and information on exposures and potential confounders  (b) Indicate the number of participants with missing data for each variable of interest  (c) *Cohort study* - summarise follow-up time (*e.g.*, average and total amount) | | a) Results, Paragraph 3/Table 1.  b) Table 1  c) Results, Paragraph 1 – “There were a total of 693,388 patients included, with median age 70.7 and 452,896 (65.5%) were men, over 1,872,468 person-years follow-up.” Methods, Paragraph 4 “This resulted in a maximum observed follow up time of 8.4-years (median and interquartile range: 2.3, 0.9–4.0 years), representing 1,872,468 person-years at risk.” |
| Outcome data | 15 | *Cohort study* - Report numbers of outcome events or summary measures over time  *Case-control study* - Report numbers in each exposure category, or summary measures of exposure  *Cross-sectional study* - Report numbers of outcome events or summary measures | | Results, Paragraph 4 “Unadjusted all-cause mortality was higher for those in class one compared with classes two and three respectively at 30 days (17.0% [95% CI 16.7-17.4%] vs. 10% [9.7-10.1%] and 7.4% [7.3-7.5%]), 1 year (39.8% [39.3-40.2%] vs. 21.4% [21.1-21.6%] and 14.4% [14.2-14.5%]) and 5 years (57.4% [57.0-57.9%] vs. 34.0% [33.6-34.3%] and 22.4% [22.3-22.6%]).” |
| Main results | 16 | (a) Give unadjusted estimates and, if applicable, confounder-adjusted estimates and their precision (e.g., 95% confidence interval). Make clear which confounders were adjusted for and why they were included  (b) Report category boundaries when continuous variables were categorized  (c) If relevant, consider translating estimates of relative risk into absolute risk for a meaningful time period | | Table 2 |
| Other analyses | 17 | Report other analyses done—e.g., analyses of subgroups and interactions, and sensitivity analyses | |  |
| **Discussion** | | | | |
| Key results | 18 | Summarise key results with reference to study objectives | | Discussion, Paragraph 1 |
| Limitations | 19 | Discuss limitations of the study, taking into account sources of potential bias or imprecision. Discuss both direction and magnitude of any potential bias | RECORD 19.1: Discuss the implications of using data that were not created or collected to answer the specific research question(s). Include discussion of misclassification bias, unmeasured confounding, missing data, and changing eligibility over time, as they pertain to the study being reported. | Discussion, Paragraph 7 - MINAP is designed to be representative of the management of acute coronary syndromes in a clinical setting and our previous work has shown consistent results to that produced by randomized clinical trial data replicated in a real world clinical setting.  Limitations are discussed in detail:  Discussion, Paragraph 7 “Our analyses are likely to have underestimated the impact of multimorbidity on survival following AMI. This is because 1) some long-term health conditions may have been under-recorded in MINAP and 2) because we did not have information about other diseases such as chronic arthritis, mental illness, dementia, obesity, cancer, and inflammatory bowel disease.”  Discussion, Paragraph 7: Despite these strengths, there were other study limitations. 1) The study was reliant upon the accurate recording of data, and MINAP does not have 100% case ascertainment. 2) Missing data, in particular missing data for each of the multimorbid conditions, could have biased the estimates. However, a thorough imputation strategy, including for multimorbid conditions, was implemented to minimise bias following a previous comprehensive study of the nature of missing data within MINAP.[36] 3) The study was limited to all-cause mortality due to the lack of available cause-specific mortality data. However, it has been shown that cause-specific mortality data may not always be reliable for cardiovascular related causes of death.[49] 4) The study includes historical data ranging from 2003 to 2013, which may therefore underestimate the most recent survival rates due to improved treatments over time. Moreover, there was an apparent increase in the number of multimorbid conditions from 2003 to 2004, which may be a result of improved recording following the introduction of the quality outcomes framework in 2004 which incentivised general practitioners to identify and screen for comorbid conditions or a result of improved case ascertainment in this time period for patients who had non-ST elevation myocardial infarction (NSTEMI) amongst whom multimorbidity tends to be more common. 5) The observational nature of the study implied we cannot demonstrate causation, though adjustment was made for confounders based on a rich set of available information in the study dataset and informed by external information from other studies. |
| Interpretation | 20 | Give a cautious overall interpretation of results considering objectives, limitations, multiplicity of analyses, results from similar studies, and other relevant evidence | | Throughout Discussion. |
| Generalisability | 21 | Discuss the generalisability (external validity) of the study results | | Discussion, Paragraph 7 “To our knowledge, MINAP is the largest nationwide single healthcare system database covering a prospective cohort of acute coronary syndromes. MINAP is designed to be representative of the management of acute coronary syndromes in a clinical setting and our previous work has shown consistent results to that produced by randomized clinical trial data replicated in a real world clinical setting.” |
| **Other information** | | | | |
| Funding | 22 | Give the source of funding and the role of the funders for the present study and, if applicable, for the original study on which the present article is based | | Following journal requirements, these have been submitted via the online submission forms to be published alongside the final published article. |

*Give information separately for cases and controls in case-control studies and, if applicable, for exposed and unexposed groups in cohort and cross-sectional studies.

Reference: Benchimol EI, Smeeth L, Guttmann A, Harron K, Moher D, Petersen I, Sørensen HT, von Elm E, Langan SM, the RECORD Working Committee. The REporting of studies Conducted using Observational Routinely-collected health Data (RECORD) Statement. PLoS Medicine 2015; in press. Checklist is protected under Creative Commons Attribution ([CC BY](http://creativecommons.org/licenses/by/4.0/)) license.
