# Supplementary figures and images for: Multimorbidity and survival for patients with acute myocardial infarction in England and Wales: Latent class analysis of a nationwide population-based cohort
Source: PLoS Med. 2018 Mar 6;15(3):e1002501. doi: 10.1371/journal.pmed.1002501 (PMC5839532; doi:10.1371/journal.pmed.1002501)

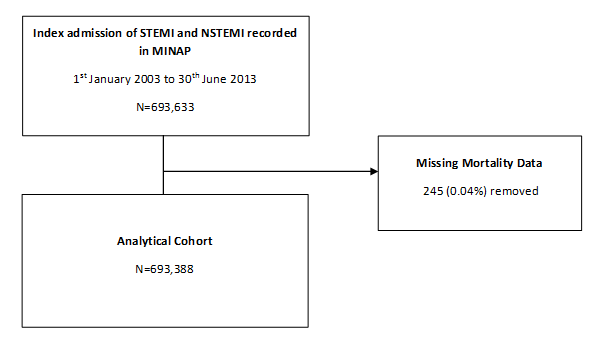

Supplement: S1 Fig — (TIF) [file pmed.1002501.s002.tif]

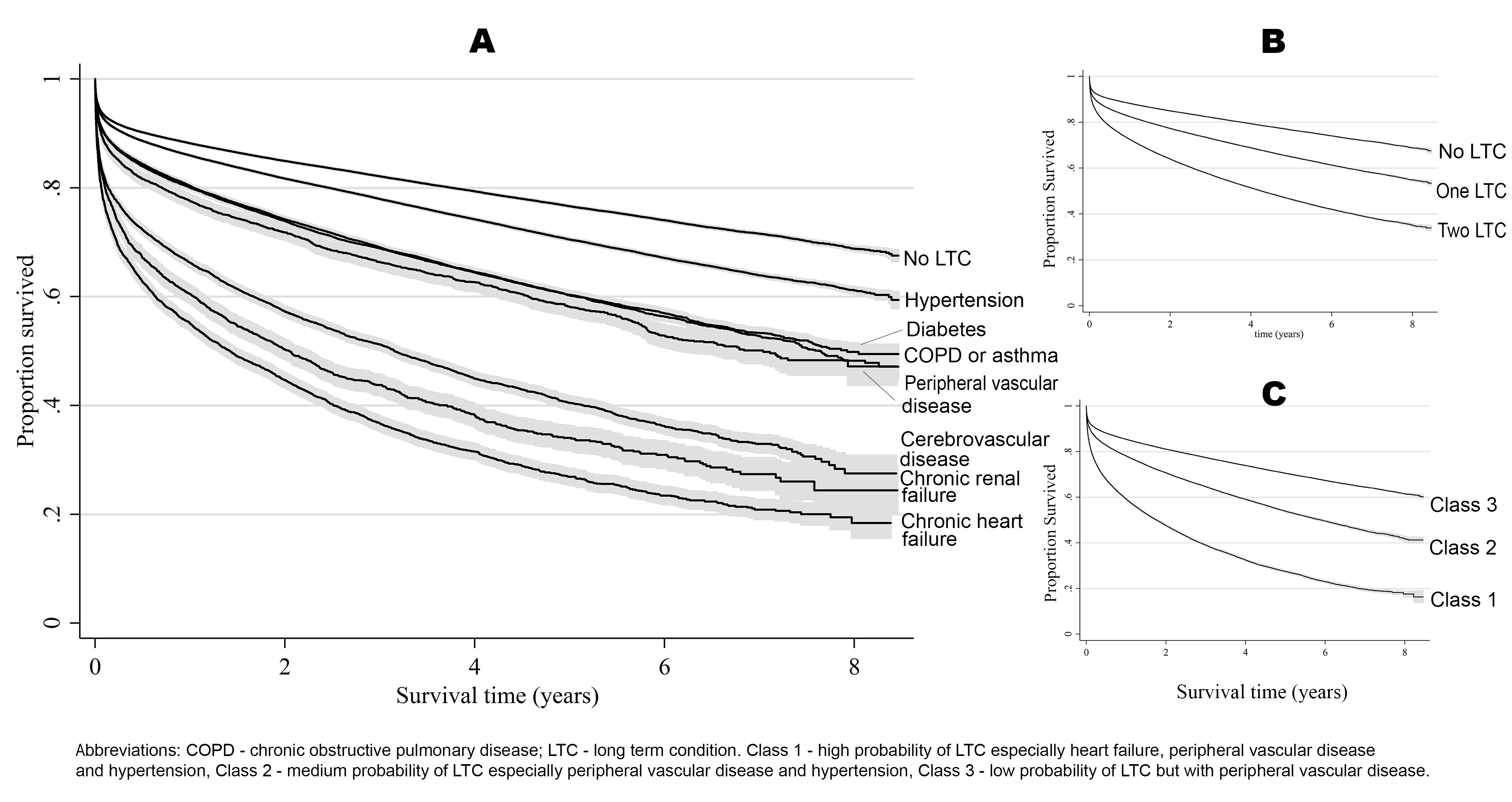

Supplement: S2 Fig — (TIF) [file pmed.1002501.s003.tif]

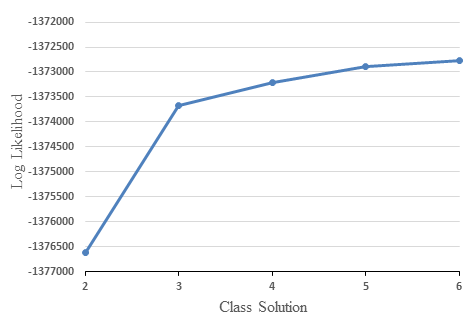

Supplement: S3 Fig — (TIF) [file pmed.1002501.s004.tif]
