# Supplementary material for: Multimorbidity and survival for patients with acute myocardial infarction in England and Wales: Latent class analysis of a nationwide population-based cohort
Source: PLoS Med. 2018 Mar 6;15(3):e1002501. doi: 10.1371/journal.pmed.1002501 (PMC5839532; doi:10.1371/journal.pmed.1002501)
Supplement: S1 Table — (DOCX) [file pmed.1002501.s005.docx]

**S1 Table.** Imputation model specification.

| **Variable** | **Variable Type** | **Imputation method** |
| --- | --- | --- |
| Age | Continuous, non-normal | Predictive mean matching |
| Index of multiple deprivation score | Continuous, non-normal | Predictive mean matching |
| Ethnicity | Categorical | Polytomous logistic regression |
| Sex | Binary | Logistic regression |
| Systolic blood pressure | Continuous, non-normal | Predictive mean matching |
| Heart rate | Continuous, non-normal | Predictive mean matching |
| Peak troponin | Continuous, non-normal | Predictive mean matching |
| Creatinine | Continuous, non-normal | Predictive mean matching |
| Loop diuretic | Binary | Logistic regression |
| Cardiac arrest | Binary | Logistic regression |
| ECG Appearance | Categorical | Polytomous logistic regression |
| Aspirin | Binary | Logistic regression |
| Beta-blocker | Binary | Logistic regression |
| Statin | Binary | Logistic regression |
| ACEi or ARBs | Binary | Logistic regression |
| P2Y_12_ inhibitors | Binary | Logistic regression |
| Aldosterone antagonist | Binary | Logistic regression |
| Coronary intervention | Categorical | Polytomous logistic regression |
| Diabetes mellitus | Binary | Logistic regression |
| Previous hypertension | Binary | Logistic regression |
| Elevated cholesterol | Binary | Logistic regression |
| Previous myocardial infarction | Binary | Logistic regression |
| Previous angina | Binary | Logistic regression |
| Peripheral vascular disease | Binary | Logistic regression |
| Cerebrovascular disease | Binary | Logistic regression |
| COPD or asthma | Binary | Logistic regression |
| Congestive renal failure | Binary | Logistic regression |
| Congestive cardiac failure | Binary | Logistic regression |
| Previous PCI | Binary | Logistic regression |
| Previous CABG | Binary | Logistic regression |
| Family history of chronic heart disease | Binary | Logistic regression |
| Smoking status | Binary | Logistic regression |
| Care by a cardiologist | Binary | Logistic regression |
| Year | Continuous | Predictor variable only |
| Admission diagnosis | Categorical | Predictor variable only |
| Timing of invasive coronary strategy | Continuous | Predictor variable only |
| Nelson-Aalen estimate of survival | Continuous | Predictor variable only |
| Censoring indicator | Binary | Predictor variable only |
