# Supplementary material for: Multimorbidity and survival for patients with acute myocardial infarction in England and Wales: Latent class analysis of a nationwide population-based cohort
Source: PLoS Med. 2018 Mar 6;15(3):e1002501. doi: 10.1371/journal.pmed.1002501 (PMC5839532; doi:10.1371/journal.pmed.1002501)
Supplement: S2 Table — (DOCX) [file pmed.1002501.s006.docx]

**S2 Table**: Complete case sensitivity analyses – unadjusted and adjusted flexible parametric survival models per condition (5 degrees of freedom, odds scale).

| **Multimorbidity group** | **Unadjusted HR (95% CI)** | **Adjusted HR (95% CI)^†^** |
| --- | --- | --- |
| **Latent class phenotype**^††^ | **P<0.001** | **P<0.001** |
| Class 1 | 4.20 (4.13-4.28) | 2.72 (2.56-2.89) |
| Class 2 | 1.80 (1.78-1.83) | 1.38 (1.31-1.45) |
| Class 3 | 1 (ref) | 1 (ref) |
|  |  |  |
| **Diabetes mellitus** | **P<0.001** | **P<0.001** |
| Yes | 1.67 (1.65-1.69) | 1.13 (1.07-1.18) |
| No | 1 (ref) | 1 (ref) |
|  |  |  |
| **COPD or asthma** | **P<0.001** | **P<0.001** |
| Yes | 1.70 (1.68-1.73) | 1.34 (1.27-1.42) |
| No | 1 (ref) | 1 (ref) |
|  |  |  |
| **Chronic heart failure** | **P<0.001** | **P<0.001** |
| Yes | 3.94 (3.86-4.02) | 2.10 (1.95-2.26) |
| No | 1 (ref) | 1 (ref) |
| ***LVEF*** |  |  |
| Moderate (30-49%) | 1.18 (1.07-1.30) | 1.68 (1.29-2.18) |
| Poor (<30%) | 1.80 (1.64-1.99) | 2.29 (1.76-2.99) |
| Good (≥50%) | 1 (ref) | 1 (ref) |
|  |  |  |
| **Chronic renal failure** | **P<0.001** | **P<0.001** |
| Yes | 3.46 (3.39-3.53) | 1.94 (1.82-2.08) |
| No | 1 (ref) | 1 (ref) |
| ***eGFR*** | **P<0.001** | **P<0.001** |
| Moderate eGFR (30-59) | 1.74 (1.58-1.91) | 1.51 (1.19-1.90) |
| Severe or very severe eGFR (<30) | 3.08 (2.80-3.39) | 2.55 (2.00-3.24) |
| Normal or mild eGFR (≥60) | 1 (ref) | 1 (ref) |
|  |  |  |
| **Cerebrovascular disease** | **P<0.001** | **P<0.001** |
| Yes | 2.70 (2.66-2.75) | 1.67 (1.58-1.77) |
| No | 1 (ref) | 1 (ref) |
|  |  |  |
| **Peripheral vascular disease** | **P<0.001** | **P<0.001** |
| Yes | 2.19 (2.14-2.24) | 1.39 (1.28-1.51) |
| No | 1 (ref) | 1 (ref) |
|  |  |  |
| **Hypertension** | **P<0.001** | **P=0.293** |
| Yes | 1.28 (1.26-1.29); | 1.02 (0.98-1.07) |
| No | 1 (ref) | 1 (ref) |
|  |  |  |
| **Cumulative conditions** | **P<0.001** | **P<0.001** |
| One | 1.70 (1.67-1.72) | 1.34 (1.27-1.41) |
| Two or more | 3.18 (3.14-3.22) | 2.11 (2.00-2.23) |
| None | 1 (ref) | 1 (ref) |
| ^†^Adjusted for sex, year of admission, index of multiple deprivation, GRACE risk score, phenotype (ST-elevation myocardial infarction vs. non-ST-elevation myocardial infarction), smoking status, family history of coronary heart disease, previous myocardial infarction, previous percutaneous coronary intervention, serum cholesterol, revascularisation (thrombolysis or coronary intervention (PCI or CABG) or both) and discharge medications (aspirin, β-blocker, ACEi/ARBs, statins, P2Y_12_ inhibitors, aldosterone antagonist). ^††^Class 1 characterises patients with high levels of long-term conditions especially chronic heart failure, peripheral vascular disease and hypertension, Class 2 characterises patients with medium levels of long-term conditions especially peripheral vascular disease and hypertension and Class 3 characterises patients with low levels of long-term conditions but with peripheral vascular disease. | | |
