# Supplementary material for: Multimorbidity and survival for patients with acute myocardial infarction in England and Wales: Latent class analysis of a nationwide population-based cohort
Source: PLoS Med. 2018 Mar 6;15(3):e1002501. doi: 10.1371/journal.pmed.1002501 (PMC5839532; doi:10.1371/journal.pmed.1002501)
Supplement: S3 Table — (DOCX) [file pmed.1002501.s007.docx]

**S3 Table**: Time period sensitivity analyse – unadjusted and adjusted flexible parametric survival models per condition (5 degrees of freedom, odds scale) for the period 2004-2013 (excluding 2003).

| **Multimorbidity group** | **Unadjusted HR (95% CI)** | **Adjusted HR (95% CI)^†^** |
| --- | --- | --- |
| **Latent class phenotype**^††^ | **P<0.001** | **P<0.001** |
| Class 1 | 4.22 (4.14-4.29) | 2.29 (2.22-2.36) |
| Class 2 | 1.81 (1.79-1.84) | 1.55 (1.51-1.60) |
| Class 3 | 1 (ref) | 1 (ref) |
|  |  |  |
| **Diabetes mellitus** | **P<0.001** | **P<0.001** |
| Yes | 1.67 (1.65-1.69) | 1.20 (1.18-1.23) |
| No | 1 (ref) | 1 (ref) |
|  |  |  |
| **COPD or asthma** | **P<0.001** | **P<0.001** |
| Yes | 1.69 (1.67-1.72) | 1.18 (1.15-1.21) |
| No | 1 (ref) | 1 (ref) |
|  |  |  |
| **Chronic heart failure** | **P<0.001** | **P<0.001** |
| Yes | 3.90 (3.82-3.98) | 1.88 (1.81-1.94) |
| No | 1 (ref) | 1 (ref) |
| ***LVEF*** | **P<0.001** | **P<0.001** |
| Moderate (30-49%) | 1.21 (1.10-1.33) | 1.27 (1.10-1.47) |
| Poor (<30%) | 1.86 (1.70-2.05) | 1.91 (1.64-2.22) |
| Good (≥50%) | 1 (ref) | 1 (ref) |
|  |  |  |
| **Chronic renal failure** | **P<0.001** | **P<0.001** |
| Yes | 3.44 (3.37-3.51) | 1.76 (1.69-1.82) |
| No |  |  |
| ***eGFR*** | **P<0.001** |  |
| Moderate (30-59) | 1.75 (1.58-1.95) | 1.51 (1.29-1.77) |
| Severe or very severe eGFR (<30) | 2.86 (2.58-3.17) | 2.12 (1.82-2.46) |
| Normal or mild eGFR (≥60) | 1 (ref) | 1 (ref) |
|  |  |  |
| **Cerebrovascular disease** | **P<0.001** | **P<0.001** |
| Yes | 2.68 (2.63-2.72) | 1.75 (1.70-1.80) |
| No | 1 (ref) | 1 (ref) |
|  |  |  |
| **Peripheral vascular disease** | **P<0.001** | **P<0.001** |
| Yes | 2.13 (2.08-2.18) | 1.39 (1.33-1.44) |
| No | 1 (ref) | 1 (ref) |
|  |  |  |
| **Hypertension** | **P<0.001** | **P<0.001** |
| Yes | 1.30 (1.28-1.31) | 1.04 (1.02-1.06) |
| No | 1 (ref) | 1 (ref) |
|  |  |  |
| **Cumulative number of conditions** | **P<0.001** | **P<0.001** |
| One | 1.71 (1.69-1.74) | 1.51 (1.47-1.56) |
| Two or more | 3.32 (3.27-3.37) | 2.56 (2.48-2.64) |
| None | 1 (ref) | 1 (ref) |
| ^†^Adjusted for sex, year of admission, index of multiple deprivation (continuous), GRACE risk score (categorised into lowest (<70), low (70 to 87) and intermediate-to-high risk (≥88)), phenotype (ST-elevation myocardial infarction vs. non-ST-elevation myocardial infarction), smoking status, family history of coronary heart disease, history of hypertension, previous myocardial infarction, previous percutaneous coronary intervention, serum cholesterol (continuous), revascularisation (thrombolysis or coronary intervention (PCI or CABG) or both) and discharge medications (aspirin, β-blocker, ACEi/ARBs, statins, P2Y_12_ inhibitors, aldosterone antagonist). ^††^Class 1 characterises patients who are highly multimorbid especially with concomitant chronic heart failure, peripheral vascular disease and hypertension, Class 2 characterises patients with medium levels of multimorbidity especially peripheral vascular disease and hypertension and Class 3 characterises patients with low levels of multimorbidity but with peripheral vascular disease. | | |
