# Supplementary material for: Multimorbidity and survival for patients with acute myocardial infarction in England and Wales: Latent class analysis of a nationwide population-based cohort
Source: PLoS Med. 2018 Mar 6;15(3):e1002501. doi: 10.1371/journal.pmed.1002501 (PMC5839532; doi:10.1371/journal.pmed.1002501)
Supplement: S4 Table — (DOCX) [file pmed.1002501.s008.docx]

**S4 Table:** Model fit statistics for latent class solutions

|  | **1** | **2** | **3** | **4** | **5** | **6** |
| --- | --- | --- | --- | --- | --- | --- |
| No of free parameters | 7 | 15 | 23 | 31 | 39 | 47 |
| Log likelihood | -1405284.609 | -1376608.851 | -1373673.359 | -1373201.448 | -1372895.880 | -1372767.032 |
| Lo-Mendell-Rubin LR test (2*Loglikelihood difference, *P*-value) | NA | 57351.514; <0.001 | 5870.984; <0.001 | 943.823, <0.001 | 611.135, <0.001 | 257.698, <0.001 |
| Parametric bootstrapped LR test,  (2*Loglikelihood difference, *P*-value) | NA | 57351.514; <0.001 | 5870.984; <0.001 | 943.823, <0.001 | 611.135, <0.001 | 257.698, <0.001 |
| Entropy | NA | 0.401 | 0.414 | 0.406 | 0.439 | 0.473 |
| Akaike’s Information Criterion | 2810583.217 | 2753247.703 | 2747392.718 | 2746464.896 | 2745869.761 | 2745628.063 |
| Bayesian Information Criterion | 2810661.967 | 2753416.452 | 2747651.468 | 2746813.645 | 2746308.510 | 2746156.812 |
| Sample size adjusted Bayesian Information Criterion | 2810639.721 | 2753368.782 | 2747578.373 | 2746715.126 | 2746184.566 | 2746007.444 |
