# Supplementary material for: Multimorbidity and survival for patients with acute myocardial infarction in England and Wales: Latent class analysis of a nationwide population-based cohort
Source: PLoS Med. 2018 Mar 6;15(3):e1002501. doi: 10.1371/journal.pmed.1002501 (PMC5839532; doi:10.1371/journal.pmed.1002501)
Supplement: S5 Table — (DOCX) [file pmed.1002501.s009.docx]

**S5 Table:** Conditional probabilities of the latent class structure.

| **Long-term condition** | **Class 1** | **Class 2** | **Class 3** |
| --- | --- | --- | --- |
| Diabetes mellitus | 0.296 | 0.016 | 0.018 |
| COPD or asthma | 0.218 | 0.125 | 0.036 |
| Chronic heart failure | 0.732 | 0.122 | 0.128 |
| Chronic renal failure | 0.267 | 0.046 | 0.004 |
| Cerebrovascular disease | 0.158 | 0.057 | 0.013 |
| Peripheral vascular disease | 0.645 | 0.789 | 0.298 |
| Hypertension | 0.415 | 0.358 | 0.065 |
| Conditional probabilities indicate the probability of a person having each respective condition given they are in Class 1, 2 or 3. | | | |
