# Supplementary material for: Multimorbidity and survival for patients with acute myocardial infarction in England and Wales: Latent class analysis of a nationwide population-based cohort
Source: PLoS Med. 2018 Mar 6;15(3):e1002501. doi: 10.1371/journal.pmed.1002501 (PMC5839532; doi:10.1371/journal.pmed.1002501)
Supplement: S6 Table — (DOCX) [file pmed.1002501.s010.docx]

**S6 Table:** Patient characteristics for those with missing latent class data (prior to multiple imputation for missing data).

| **Variable** |  | **N=125,325** | **Missing (%)** |
| --- | --- | --- | --- |
| Age (years) | Median (IQR) | 70.7 (59.5-80.0) | 0.4% |
| Sex (male) | N (%) | 81,922 (65.7) | 0.6% |
| Year of admission |  |  | 0% |
| 2003-2006 | N (%) | 65,926 (52.6) |  |
| 2007-2010 | N (%) | 40,274 (32.1) |  |
| 2011-2013 | N (%) | 19,125 (15.3 |  |
| Deprivation | Median (IQR) | 19.2 (11.2-33.7) | 0.1% |
| GRACE risk score |  |  | 85.4% |
| ≤70 | N (%) | 1115 (6.1%) |  |
| 71-87 | N (%) | 1639 (8.9%) |  |
| ≥88 | N (%) | 15,499 (84.9%) |  |
| Index AMI |  |  |  |
| STEMI vs. NSTEMI | N (%) | 57,879 (46.2%) | 0% |
| SBP (mm Hg) | Mean (sd) | 138.9 (30.0) | 56.5% |
| Heart rate (beat/min) | Mean (sd) | 82.4 (24.3) | 56.5% |
| Total cholesterol (mg/dL) | Median (IQR) | 196.6 (55.7) | 41.2% |
| Creatinine (mg/dL) | Median (IQR) | 1.09 (0.89-1.35) | 73.1% |
| Medical history |  |  |  |
| Current or ex-smoker | N (%) | 53,043 (57.3%) | 26.1% |
| Family history of coronary heart disease | N (%) | 11,472 (39.6%) | 76.9% |
| Previous acute myocardial infarction | N (%) | 15,711 (27.5%) | 54.4% |
| Previous angina | N (%) | 18,312 (36.1%) | 59.6% |
| Admission treatment |  |  |  |
| Revascularisation^§§^ | N (%) | 57,937 (61.2%) | 24.5% |
| Loop diuretic | N (%) | 12,667 (31.2%) | 67.6% |
| Discharge medication |  |  |  |
| Aspirin | N (%) | 85,471 (79.9%) | 14.7% |
| β-blocker | N (%) | 69,016 (71.4%) | 22.9% |
| Statin | N (%) | 82,655 (76.5%) | 13.7% |
| ACEi or ARB | N (%) | 71,837 (69.6%) | 17.6% |
| P2Y_12_ inhibitor | N (%) | 24,184 (91.1%) | 78.8% |
| Aldosterone antagonist | N (%) | 1,729 (10.1%) | 86.4% |
| Mortality (unadjusted) |  |  |  |
| 30-Day | N (%) | 14,153 (11.3%) | 0% |
| 1 year | N (%) | 25,387 (20.3%) | 0% |
| 5 years | N (%) | 40,412 (32.3%) | 0% |
| §§Thrombolysis or coronary intervention (PCI or CABG) or both. ††Given international guidelines, GRACE risk score categories were categorised into lowest (<70), low (70 to 87) and intermediate-to-high risk (≥88). Abbreviations: ARB - Angiotensin receptor blocker; ACEi - angiotensin-converting enzyme inhibitor; SBP – systolic blood pressure; NSTEMI – non ST-elevated myocardial infarction; STEMI - ST-elevated myocardial infarction. | | | |
