# Supplementary material for: Multimorbidity and survival for patients with acute myocardial infarction in England and Wales: Latent class analysis of a nationwide population-based cohort
Source: PLoS Med. 2018 Mar 6;15(3):e1002501. doi: 10.1371/journal.pmed.1002501 (PMC5839532; doi:10.1371/journal.pmed.1002501)
Supplement: S7 Table — (DOCX) [file pmed.1002501.s011.docx]

**S7 Table:** Loss of life expectancy and 95% confidence intervals by long-term condition and age at hospitalisation for patients with acute myocardial infarction between 2003 and 2013 compared with the age, sex and year matched population of England and Wales.

|  | **Age at time of acute myocardial infarction hospitalisation** | | | |
| --- | --- | --- | --- | --- |
|  | **60** | **70** | **80** | **All Ages** |
| **Population life expectancy (mean (SD) in years)*** | +22.2 (2.0) | +14.8 (1.5) | +8.6 (0.9) | +16.6 (9.8) |
| **Cohort life expectancy (mean (95% CI) in years)** | +20.6 (20.4-20.8) | +13.2 (13.0-13.4) | +7.39 (7.26-7.52) | +17.4 (17.2-17.5) |
| **Cohort loss of life expectancy (mean (95% CI) in years)** | 1.55 (1.35-1.75) | 1.65 (1.46-1.84) | 1.23 (1.10-1.36) | 1.14 (0.99-1.28) |
|  |  |  |  |  |
| **Loss of life expectancy by condition (mean (95% CI) in years)** | | | |  |
|  | **60** | **70** | **80** | **All Ages** |
| Diabetes mellitus | 1.99 (1.70-2.27) | 2.47 (2.19-2.74) | 1.48 (1.31-1.65) | 1.82 (1.59-2.05) |
| COPD or asthma | 2.18 (1.87-2.50) | 2.13 (1.86-2.40) | 1.62 (1.43-1.80) | 1.74 (1.51-1.97) |
| Chronic heart failure | 5.12 (4.39-5.85) | 3.95 (3.46-4.44) | 2.79 (2.50-3.09) | 2.91 (2.58-3.25) |
| Chronic renal failure | 5.18 (4.63-5.74) | 4.46 (4.02-4.89) | 2.23 (1.98-2.49) | 2.78 (2.47-3.10) |
| Cerebrovascular disease | 3.44 (3.04-3.83) | 3.40 (3.03-3.77) | 1.93 (1.71-2.15) | 2.09 (1.84-2.34) |
| Peripheral vascular disease | 2.48 (2.11-2.84) | 2.06 (1.76-2.37) | 1.63 (1.42-1.84) | 2.14 (1.86-2.42) |
| Hypertension | 1.58 (1.36-1.80) | 1.81 (1.61-2.04) | 1.31 (1.17-1.46) | 0.72 (0.64-0.80) |
|  |  |  |  |  |
| Cumulative effect |  |  |  |  |
| One (vs. none) | 1.58 (1.37-1.80) | 1.44 (1.27-1.61) | 1.11 (0.99-1.23) | 1.03 (0.89-1.16) |
| Two or more (vs. none) | 2.17 (1.87-2.46) | 2.62 (2.35-2.89) | 1.65 (1.49-1.82) | 1.86 (1.64-2.08) |
|  |  |  |  |  |
| Multiple conditions (latent classes structure)^††^ |  |  |  |  |
| Class 1 (vs. class 3) | 5.42 (4.84-6.00) | 3.87 (3.45-4.30) | 2.26 (2.04-2.48) | 2.89 (2.59-3.19) |
| Class 2 (vs. class 3) | 1.61 (1.38-1.84) | 2.29 (2.05-2.53) | 1.41 (1.26-1.56) | 1.52 (1.33-1.71) |
| *Age, sex and year matched population life expectancy of England and Wales according to office for national statistics lifetables, conditional to getting to age 60, 70 or 80 respectively. ^††^Class 1 characterises patients who are highly multi-morbid especially with concomitant chronic heart failure, peripheral vascular disease and hypertension, Class 2 characterises patients with medium levels of multi-morbidity especially peripheral vascular disease and hypertension and Class 3 characterises patients with low levels of multi-morbidity but with peripheral vascular disease. | | | | |
