# Supplementary material for: Multimorbidity and survival for patients with acute myocardial infarction in England and Wales: Latent class analysis of a nationwide population-based cohort
Source: PLoS Med. 2018 Mar 6;15(3):e1002501. doi: 10.1371/journal.pmed.1002501 (PMC5839532; doi:10.1371/journal.pmed.1002501)
Supplement: S1 Text — (DOCX) [file pmed.1002501.s012.docx]

**S1 Text:** Data access and data cleaning

The authors had access to all admissions recorded in MINAP in the period 1^st^ January 2003 to 30^th^ June 2013. The authors only had access to anonymised patient level data, which were linked to the Office for National Statistics death records prior to data release to the authors. The authors had access to mortality status and follow-up/survival times, rather than full dates of death or follow-up. Data cleaning was undertaken on the full population based dataset, from which duplicate records were removed, as well as cases where the arrival month and year were missing. Cases for which derived survival/follow-up times were negative were set to missing. Derivation of the analytical cohort of index admissions of STEMI and NSTEMI is presented in S1 Fig.
