# Supplementary material for: Multimorbidity and survival for patients with acute myocardial infarction in England and Wales: Latent class analysis of a nationwide population-based cohort
Source: PLoS Med. 2018 Mar 6;15(3):e1002501. doi: 10.1371/journal.pmed.1002501 (PMC5839532; doi:10.1371/journal.pmed.1002501)
Supplement: S2 Text — (DOCX) [file pmed.1002501.s013.docx]

**S2 Text**: Multiple Imputation Analysis

Multiple imputation by chained equations (MICE) was used to generate ten imputed datasets using 20 iterations. The imputation process was stratified according to AMI phenotype (according to ST-elevation myocardial infarction and non ST-elevation myocardial infarction) to allow for important different relationships between variables for each phenotype to be taken into account. The imputation strategy details are provided in S1 Table.

As per multiple imputation good practice guidelines,[[1](#_ENREF_1)] a sensitivity of the main analysis results, which accounted for missing data using multiple imputation, were compared to a complete case analysis. S2 Fig and S2 Table contain the complete case analysis equivalent results to the main analysis shown in manuscript Fig 3 and Table 2.

**References**

1. White IR, Royston P, Wood AM (2011) Multiple imputation using chained equations: issues and guidance for practice. Stat Med 30: 377-399.
