# Supplementary material for: Multimorbidity and survival for patients with acute myocardial infarction in England and Wales: Latent class analysis of a nationwide population-based cohort
Source: PLoS Med. 2018 Mar 6;15(3):e1002501. doi: 10.1371/journal.pmed.1002501 (PMC5839532; doi:10.1371/journal.pmed.1002501)
Supplement: S3 Text — (DOCX) [file pmed.1002501.s014.docx]

**S3 Text:** Latent class analysis

Latent class analysis is a modelling based approach used to classify cases into unobserved groupings based on similarity of patterns across multivariate data.[[1](#_ENREF_1)] These unobserved groupings are called latent classes, and the resulting classes can represent complex interactions across multivariate data. In our study, latent classes were determined based on the similarity of cases according to their presence or absence of multimorbidity. Other techniques which try to look at interacting effects of multimorbidity include consideration of all possible combinations of conditions (however, this results in a high Type I error rate and low study power if there are few patients within each combination), and clustering analyses (which although offering insights into how data are clustered, cannot provide further detail on the probabilities of class membership and rely on arbitrary measures of distance between data points, rather than accounting for the underlying data distributions).

Latent classes were derived using a mixture modelling approach in Mplus.[[2](#_ENREF_2),[3](#_ENREF_3)] We explored several class solutions starting with a two class model and subsequently increasing the number of classes up to a maximum of 6 classes. S4 Table summarises the model fit statistics of the class solutions explored. The preferred solution was selected based on the interpretability of the emerging classes, minimisation of the Bayesian information criteria (BIC),[[4](#_ENREF_4)] and the class solution where the log likelihood plot started to level off (S3 Fig). We evaluated classification quality using the entropy statistic. The entropy statistic is calculated from the posterior probabilities, it ranges from 0 to 1 and higher values indicate that the latent class is more distinct.[[5](#_ENREF_5)] Although the lowest BIC was achieved by the 6 class model (S4 Table), when looking at the entropy, the log likelihood plot and the clinical interpretability of the emerging classes, the 3 class solution provided the optimal solution. Gains in model fit beyond the 3 class solution were minimal (S3 Fig).

The conditional probabilities of the latent class structure for the final three class solution are provided in S5 Table, and the clinical and demographic variables according to class membership are provided in S6 Table.

**References**

1. Hagenaars JA, McCutcheon AL (2002) Applied latent class analysis: Cambridge University Press.

2. Muthén B, Muthén L (2013) Software Mplus Version 7.31.

3. Henry KL, Muthén B (2010) Multilevel latent class analysis: An application of adolescent smoking typologies with individual and contextual predictors. Struct Equ Modeling 17: 193-215.

4. Konishi S, Kitagawa G (2008) Bayesian information criteria. Information Criteria and Statistical Modeling: 211-237.

5. Pastor DA, Barron KE, Miller B, Davis SL (2007) A latent profile analysis of college students’ achievement goal orientation. Contemp Educ Psychol 32: 8-47.
