# Supplementary material for: Multimorbidity and survival for patients with acute myocardial infarction in England and Wales: Latent class analysis of a nationwide population-based cohort
Source: PLoS Med. 2018 Mar 6;15(3):e1002501. doi: 10.1371/journal.pmed.1002501 (PMC5839532; doi:10.1371/journal.pmed.1002501)
Supplement: S4 Text — (DOCX) [file pmed.1002501.s015.docx]

**S4 Text:** Loss of Life Expectancy

Loss in life expectancy represents the difference between the expected remaining lifetime in a disease free population and the expected remaining lifetime in the diseased population. Loss in life expectancy was calculated using the approach defined by Andersson *et al* (2013) and based on Royston-Parmar relative survival models. The mortality of the cohort was compared with that of the mortality in an age, sex and year matched general population. This allows the estimation of the likelihood that a patient will die from causes associated with acute myocardial infarction. Therefore, this technique overcomes a lack of availability of cause-specific death data. Further details of relative survival can be found in our previous publications.[[1](#_ENREF_1),[2](#_ENREF_2)]

**References**

1. Hall M, Alabas OA, Dondo TB, Jernberg T, Gale CP (2015) Use of relative survival to evaluate non-ST-elevation myocardial infarction quality of care and clinical outcomes. Eur Heart J Quality of Care and Clinical Outcomes 1: 85-91.

2. Alabas O, Hall M, Dondo T, Rutherford MJ, Timmis A, et al. (2016) Long term excess mortality associated with diabetes following acute myocardial infarction: A population-based cohort study. Journal of Epidemiology and Community Health
